# Circadian Genotypes in *PER3* and Time of Prostate Cancer Radiotherapy Interact to Affect Risk of Late Side Effects

**DOI:** 10.1158/1078-0432.CCR-26-0268

**Published:** 2026-07-13

**Authors:** Adam J. Webb, Emily Harper, Tim Rattay, Miguel E. Aguado-Barrera, Barbara Avuzzi, Renée Bultijnck, Ananya Choudhury, Dirk K. De Ruysscher, Olivia Fuentes-Ríos, Sara Gutiérrez-Enríquez, Sarah L. Kerns, Ramón Lobato-Busto, Alan McWilliam, Barbara Noris Chiorda, Tiziana Rancati, Barry S. Rosenstein, Petra Seibold, Elena Sperk, Ana Vega, Liv Veldeman, Tim H. Ward, Catharine M. West, Paul Symonds, Christopher J. Talbot, the REQUITE Consortium, Rita Aerts, Soumia Arredouani, David Azria, Celine Bourgier, Muriel Brengues, Erik Briers, Patricia Calvo-Crespo, Ana Carballo, Angel Carracedo, Jenny Chang-Claude, Ana Crujeiras González, Wilfried De Neve, Gilles Defraene, Pieter Deseyne, Hannah Dobbelaere, Isabel Dominguez-Rios, Roxana Draghici, Alison Dunning, Fréderic Duprez, Michael Ehmann, Elhaseen Elhamin, Rebecca M. Elliott, Vicki Emms, Laura Fachal, Corinne Faivre-Finn, Irene Fajardo-Paneque, Marie-Pierre Farcy-Jacquet, Juan Fernández-Tajes, Valérie Fonteyne, Marzia Franceschini, Benjamin Gauter-Fleckenstein, Frank Giordano, Alexandra Giraldo, Antonio Gómez-Caamaño, Karin Haustermans, Irmgard Helmbold, Carsten Herskind, Philipp Heumann, Kerstie Johnson, Maarten Lambrecht, Samuel Lavers, Yolande Lievens, Marcus Mareel, Monserrat Miranda Currás, Meritxell Mollà, Christel Monten, Anusha Müller, Piet Ost, Leen Paelinck, Stephanie Peeters, Paula Peleteiro, Giselle Post, Mónica Ramos, Belinda Rodríguez-Lage, Paloma Sosa-Fajardo, Hilary Stobart, Martijn Swimberghe, Begoña Taboada-Valladares, Laura Torrado-Moya, Marc van Eijkeren, Annick Van Greveling, Erik van Limberghen, Katrien Vandecasteele, Ben Vanneste, Marlon Veldwijk, Tom Vercauteren, Caroline Weltens

**Affiliations:** 1 Division of Genetics and Genome Biology, College of Life Sciences, University of Leicester, Leicester, United Kingdom.; 2 Leicester Cancer Research Centre, College of Life Sciences, University of Leicester, Leicester, United Kingdom.; 3 Aston University, Birmingham, United Kingdom.; 4 Leicester Cancer Research Centre, University of Leicester, Leicester Royal Infirmary, Leicester, United Kingdom.; 5 Instituto de Investigación Sanitaria de Santiago de Compostela (IDIS), Santiago de Compostela, Spain.; 6 Fondazione IRCCS Istituto Nazionale Tumori, Milan, Italy.; 7 Department of Human Structure and Repair, Ghent University, Ghent, Belgium.; 8 Department of Radiation Oncology, Ghent University Hospital, Ghent, Belgium.; 9 University of Manchester, The Christie NHS Foundation Trust, Manchester, United Kingdom.; 10 Department Radiation Oncology (Maastro), Maastricht University Medical Center+, GROW School, Maastricht, the Netherlands.; 11 Hereditary Cancer Genetics Group, Vall d’Hebron Institute of Oncology (VHIO), Vall d’Hebron Barcelona Hospital Campus, Cellex Center, Barcelona, Spain.; 12 The Medical College of Wisconsin, Milwaukee, Wisconsin.; 13 Department of Medical Physics, Hospital Clínico Universitario de Santiago de Compostela, Servizo Galego de Saúde (SERGAS), Santiago de Compostela, Spain.; 14 The University of Manchester, Manchester, United Kingdom.; 15 Fondazione IRCCS Istituto Nazionale dei Tumori, Radiotherapy Department, Milan, Italy.; 16 Data Science Unit, Fondazione IRCCS Istituto Nazionale dei Tumori, Milan, Italy.; 17 Icahn School of Medicine at Mount Sinai, New York, New York.; 18 German Cancer Research Center (DKFZ), Heidelberg, Germany.; 19 Mannheim Cancer Center, Medical Faculty Mannheim, Heidelberg University, Mannheim, Germany.; 20 Fundación Pública Galega de Medicina Xenómica (FPGMX), Servicio Galego de Saúde (SERGAS), Santiago de Compostela, Spain.; 21 Centro de Investigación en Red de Enfermedades Raras (CIBERER), Instituto de Salud Carlos III, Madrid, Spain.; 22 Ghent University Hospital /Ghent University, Ghent, Belgium.; 23 Cancer Research Advocate Forum UK, Manchester, United Kingdom.; 24 Translational Radiobiology Group, Division of Cancer Sciences, The University of Manchester, Manchester Academic Health Science Centre, Christie Hospital, Manchester, United Kingdom.

## Abstract

**Purpose:**

We showed previously that patients with breast cancer with certain circadian rhythm genotypes have higher incidences of toxicity when treated with radiotherapy at different times of day. This opens the way to genetically guided chronomodulation as a cost-effective way to reduce side effects while preserving treatment efficacy. We aimed to test whether similar time-of-day effects are observed in prostate cancer.

**Experimental Design:**

The analysis used the multinational, prospective observational REQUITE prostate cancer cohort (*n* = 1,760). All patients received external beam radiotherapy and were followed for 2 years.

Regression analyses were performed in patients with complete data, including genotypes and radiotherapy fraction times (*n* = 877). Least absolute shrinkage and selection operator (LASSO) and logistic regression models incorporated genotypes of single-nucleotide polymorphisms in circadian rhythm genes, time of radiotherapy, and their interaction. Primary endpoints were late rectal bleeding (RB) and urinary incontinence (UI).

**Results:**

Multivariable models show significant effects of genotype, treatment timing, and the interaction between PER3 genotypes and treatment timing for both primary endpoints. The 44% of patients with the rs696305 C/C genotype are predicted to reduce their risk of RB from 11% when treated at 9:00 am to 5% when treated at 5:00 pm. Similarly, the analysis predicts a significant reduction in the risk of UI from 15% to 5% by avoiding treatment in the middle of the day for patients heterozygous for rs172933 (36% of patients).

**Conclusions:**

These results agree with the earlier findings in patients with breast cancer who have undergone radiotherapy. The interaction between genotype and time of treatment suggests that groups of patients could reduce side effects and improve their quality of life through chronomodulation.

**Translational Relevance**

In this study, we examined late side effects from prostate cancer radiotherapy [rectal bleeding and urinary incontinence (UI)] with respect to both the timing of external beam radiotherapy treatment and genotypes in genes with a known circadian effect. As with our previous study in patients with breast cancer, we find that subgroups of patients present differing risk profiles dependent on both genotype in the circadian gene *PER3* and the time at which radiotherapy was delivered. Our results show that the risk of developing late side effects could be mitigated through simple and cost-effective genetically informed scheduling of radiotherapy treatment; for example, 36% of patients with prostate cancer could reduce their risk of late UI from 15% to 5% by scheduling their treatment away from midday. Other groups could see similar reductions through careful scheduling of treatments, dependent on *PER3* genotypes.

## Introduction

Prostate cancer is the second most common male cancer globally (after lung cancer), with 1.4 million cases per year, and the most common in northern European countries (83 cases per 100k; ref. 1). Although incidence increased in the UK by 50% over the past 30 years, 5- and 10-year survival rates also increased (to 86.3% and 77.6%, respectively; ref. 2). With improved survival, quality of life after treatment is increasingly important.

In the UK, 30% of patients with prostate cancer undergo radiotherapy, and 15% undergo surgery (2). Early side effects of radiotherapy are common, especially diarrhea, increased urinary frequency (UF), and urgency. Prevalence depends on the irradiated volume, radiation dose, and medication, with side effects usually settling within 4 to 8 weeks of treatment. Late side effects, however, may affect quality of life indefinitely, especially if surgery and radiotherapy are combined. Survival following surgery or radiotherapy is similar (3), but the pattern of long-term side effects differs (4). Late side effects include urinary incontinence (UI), requiring the patient to wear an incontinence pad, and rectal bleeding (RB), which in most cases is due to postradiotherapy telangiectasia of the bowel mucosa. The latter can be frightening to many patients who may interpret this as a sign of cancer recurrence or a secondary cancer.

As the risk of late long-term side effects after radical treatment may profoundly influence individual choice of treatment modality, there is a need to identify approaches to reduce their risk. One emerging approach involves adjusting the timing of treatment administration in concert with a patient’s circadian rhythm—the 24-hour-long cycles that regulate many biological processes, including those relevant to radiotherapy side effects such as cellular proliferation, DNA repair, and apoptosis (5). A central molecular clock in the suprachiasmatic nucleus of the brain responds to external triggers (zeitgebers) such as light, exercise, and nutritional intake, regulating downstream autonomous tissue-specific peripheral clocks. Both central and peripheral clocks function by a negative feedback loop involving the CLOCK and BMAL1 proteins, which activate the period (*PER*) and cryptochrome (*CRY*) genes as well as many other genes under circadian control.

We showed previously that treatment time and circadian genotype interact to affect the risk of late side effects following radiotherapy for breast cancer (6). An interaction exists between time and the genotypes of unlinked circadian rhythm genes (*CLOCK*, *PER3*, *RASD1*), with the peak time for side effects determined by genotype. Other studies, not incorporating genetics, showed worse gastrointestinal toxicity in patients with prostate cancer given radiotherapy in the evening compared with daytime (7).

Genes in the present study were selected based on previous associations with treatment time-dependent radiotherapy side effects [*CLOCK*, *PER3*, *RASD1*, and *NOCT* (6, 8)] or statistical significance in a large genome-wide association study for “morningness” (*HCRTR2* and *RASD1*; ref. 9). There is additional evidence for their biological role: *CLOCK* and *PER3* are well-established circadian genes; *NOCT* is similar to the *Xenopus laevis* core circadian gene *Nocturin*; *HCRTR2* is associated with narcolepsy and hyposomnia; and mice lacking *Rasd1* show reduced sensitivity to light and impaired synchronization to light–dark cycles (10).

The aim of this study, using data collected prospectively in the international, multicenter REQUITE study (11), was to see if the timing of prostate radiotherapy and its combination with common variants of circadian genes influenced the risk of late radiation side effects, particularly RB and UI.

## Materials and Methods

### Study participants and data source

**Figure 1.**
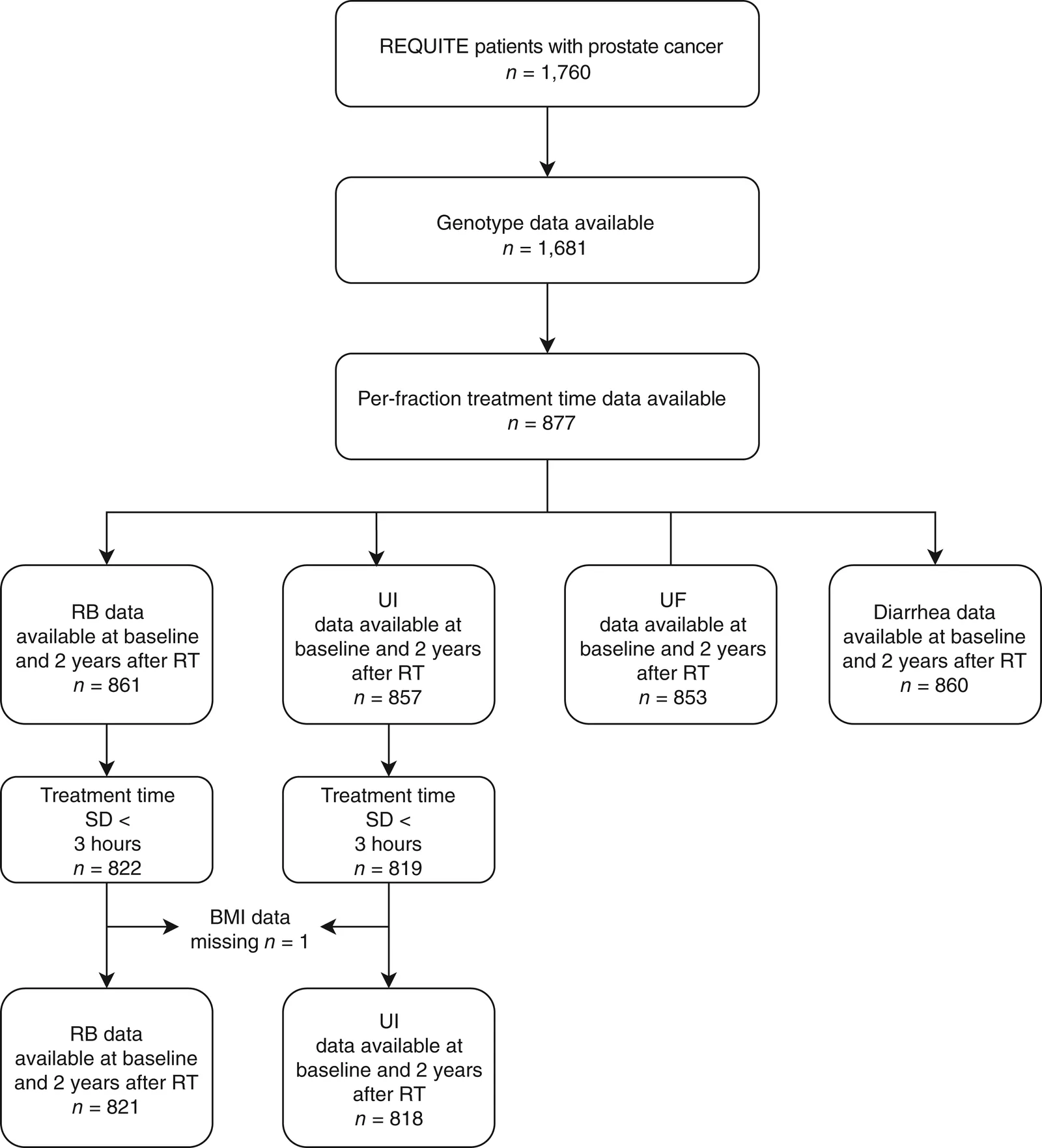
STROBE diagram showing the selection of patient cohorts for the analysis of late RB and UI. RT, radiotherapy.

Participants were drawn from the REQUITE prospective observational study (https://requite.eu), which included patients with prostate cancer receiving external beam radiotherapy recruited across 8 countries (Belgium, France, Germany, Italy, the Netherlands, Spain, the United Kingdom, and the United States) between April 2014 and March 2017. Patients with prostate cancer (*n* = 1,760) were recruited, with pretreatment, treatment (including CT imaging and radiotherapy planning data), and follow-up data collected using longitudinal standardized radiotherapy toxicity [Common Terminology Criteria for Adverse Events (CTCAE) v4.0 healthcare professional and patient-reported symptoms at the time of visit], quality of life, and treatment outcome (including physician-reported death) electronic case report forms. DNA samples were stored in a centralized biobank. Patients were followed up shortly after the completion of radiotherapy, 12 and 24 months after radiotherapy, and—optionally—every 12 months thereafter. Further details on the REQUITE study are available elsewhere (11). Of the 1,760 patients with prostate cancer recruited overall, 877 from 6 treatment centers (Table 1; Supplementary Table S1) had the requisite genotyping and detailed (per-fraction) radiotherapy treatment time-of-day-and-date data, as well as pretreatment, treatment (including CT imaging and radiotherapy planning data), and follow-up data (Fig. 1).

**Table 1.** Cohort characteristics and univariate analysis.

Characteristic	RB (late)			Diarrhea (grade 2+; late)			UF (late)			UI (late)		
	0 *n* = 773^a^	1 *n* = 88^a^	*P* value^b^	0 *n* = 852^a^	1 *n* = 8^a^	*P* value^b^	0 *n* = 694^a^	1 *n* = 159^a^	*P* value^b^	0 *n* = 726^a^	1 *n* = 131^a^	*P* value^b^
Height, cm	173 (167, 178)	173 (169, 178)	>0.9	173 (167, 178)	173 (167, 180)	>0.9	173 (167, 178)	173 (168, 178)	0.14	173 (167, 178)	175 (168, 180)	0.10
Weight, kg	82 (74, 90)	77 (70, 88)	0.005	81 (74, 90)	83 (70, 88)	0.7	81 (73, 90)	83 (74, 93)	0.037	81 (73, 90)	84 (76, 92)	0.049
Unknown	1	0	​	1	0	​	1	0	​	0	1	​
BMI, kg/m^2^	27.3 (25, 29.8)	25.9 (24.1, 28.7)	0.002	27.2 (24.9, 29.8)	26.1 (25.3, 28.5)	0.5	27.1 (24.9, 29.7)	27.6 (24.7, 30.2)	0.4	27.1 (24.8, 29.8)	27.7 (25.4, 29.7)	0.3
Unknown	1	0	​	1	0	​	1	0	​	0	1	​
Age at radiotherapy start	70 (65, 74)	72 (67, 75)	0.045	70 (65, 74)	68 (64, 73)	0.6	70 (65, 74)	70 (66, 74)	0.5	70 (65, 74)	69 (65, 74)	0.5
Smoker	​	​	0.066	​	​	0.12	​	​	0.2	​	​	0.2
0 = Never	301 (39%)	40 (45%)	​	338 (40%)	1 (13%)	​	280 (40%)	55 (35%)	​	295 (41%)	42 (32%)	​
1 = Ex before cancer diagnosis	356 (46%)	43 (49%)	​	394 (46%)	5 (63%)	​	311 (45%)	85 (53%)	​	331 (46%)	67 (51%)	​
2 = Ex since cancer diagnosis	23 (3%)	2 (2.3%)	​	24 (2.8%)	1 (13%)	​	23 (3.3%)	2 (1.3%)	​	22 (3%)	3 (2.3%)	​
3 = Current	92 (12%)	3 (3.4%)	​	95 (11%)	1 (13%)	​	79 (11%)	17 (11%)	​	77 (11%)	19 (15%)	​
7 = Do not wish to answer	0 (0%)	0 (0%)	​	0 (0%)	0 (0%)	​	0 (0%)	0 (0%)	​	0 (0%)	0 (0%)	​
Unknown	1	0	​	1	0	​	1	0	​	1	0	​
Ethnicity	​	​	>0.9	​	​	>0.9	​	​	0.6	​	​	>0.9
1 = White (European or American European)	765 (99%)	87 (100%)	​	843 (99%)	8 (100%)	​	685 (99%)	159 (100%)	​	719 (99%)	129 (99%)	​
Other	6 (0.8%)	0 (0%)	​	6 (0.7%)	0 (0%)	​	6 (0.9%)	0 (0%)	​	5 (0.7%)	1 (0.8%)	​
Unknown	2	1	​	3	0	​	3	0	​	2	1	​
MRI T stage	​	​	0.3	​	​	0.7	​	​	0.13	​	​	0.8
T2a	55 (18%)	7 (18%)	​	62 (18%)	0 (0%)	​	55 (20%)	6 (10%)	​	54 (18%)	8 (19%)	​
T2b	31 (10%)	4 (10%)	​	35 (10%)	0 (0%)	​	29 (10%)	6 (10%)	​	32 (11%)	3 (7%)	​
T2c	54 (18%)	4 (10%)	​	56 (17%)	1 (33%)	​	50 (18%)	6 (10%)	​	48 (16%)	8 (19%)	​
T3a	81 (27%)	14 (36%)	​	92 (27%)	2 (67%)	​	70 (25%)	24 (40%)	​	81 (27%)	13 (30%)	​
T3b	60 (20%)	4 (10%)	​	64 (19%)	0 (0%)	​	52 (19%)	12 (20%)	​	58 (20%)	6 (14%)	​
T4	3 (1%)	1 (2.6%)	​	4 (1.2%)	0 (0%)	​	4 (1.4%)	0 (0%)	​	4 (1.3%)	0 (0%)	​
Not visible	20 (6.6%)	5 (13%)	​	25 (7.4%)	0 (0%)	​	19 (6.8%)	6 (10%)	​	20 (6.7%)	5 (12%)	​
Unknown	469	49	​	514	5	​	415	99	​	429	88	​
cN stage	​	​	0.8	​	​	0.7	​	​	0.5	​	​	0.2
0 = *N*0	596 (77%)	71 (81%)	​	659 (77%)	6 (75%)	​	539 (78%)	120 (75%)	​	558 (77%)	104 (79%)	​
1 = *N*1	28 (3.6%)	2 (2.3%)	​	30 (3.5%)	0 (0%)	​	22 (3.2%)	8 (5%)	​	23 (3.2%)	7 (5.3%)	​
2 = NX	149 (19%)	15 (17%)	​	163 (19%)	2 (25%)	​	133 (19%)	31 (19%)	​	145 (20%)	20 (15%)	​
pN stage	​	​	0.4	​	​	0.003	​	​	0.2	​	​	<0.001
0 = *N*0	134 (37%)	7 (25%)	​	139 (36%)	2 (40%)	​	113 (36%)	26 (37%)	​	107 (33%)	34 (49%)	​
1 = *N*1	41 (11%)	4 (14%)	​	42 (11%)	3 (60%)	​	32 (10%)	12 (17%)	​	31 (9.6%)	14 (20%)	​
2 = NX	188 (52%)	17 (61%)	​	206 (53%)	0 (0%)	​	173 (54%)	33 (46%)	​	184 (57%)	22 (31%)	​
Unknown	410	60	​	465	3	​	376	88	​	404	61	​
Gleason score (biopsy)	​	​	0.3	​	​	0.7	​	​	0.2	​	​	0.2
2	1 (0.1%)	0 (0%)	​	1 (0.1%)	0 (0%)	​	1 (0.1%)	0 (0%)	​	0 (0%)	1 (0.8%)	​
4	2 (0.3%)	0 (0%)	​	2 (0.2%)	0 (0%)	​	2 (0.3%)	0 (0%)	​	2 (0.3%)	0 (0%)	​
5	9 (1.2%)	0 (0%)	​	9 (1.1%)	0 (0%)	​	8 (1.2%)	1 (0.6%)	​	7 (1%)	2 (1.5%)	​
6	139 (18%)	12 (14%)	​	150 (18%)	1 (13%)	​	126 (18%)	25 (16%)	​	133 (18%)	17 (13%)	​
7	439 (57%)	46 (52%)	​	480 (56%)	4 (50%)	​	397 (57%)	82 (52%)	​	408 (56%)	75 (57%)	​
8	98 (13%)	16 (18%)	​	112 (13%)	2 (25%)	​	88 (13%)	26 (16%)	​	98 (14%)	16 (12%)	​
9	81 (11%)	13 (15%)	​	93 (11%)	1 (13%)	​	70 (10%)	22 (14%)	​	74 (10%)	19 (15%)	​
10	2 (0.3%)	1 (1.1%)	​	3 (0.4%)	0 (0%)	​	1 (0.1%)	2 (1.3%)	​	2 (0.3%)	1 (0.8%)	​
Unknown	2	0	​	2	0	​	1	1	​	2	0	​
PSA prediagnostic biopsy, ng/mL	9 (6, 16)	10 (6, 15)	0.8	9 (6, 16)	19 (8, 58)	0.10	9 (6, 16)	9 (7, 17)	>0.9	9 (6, 16)	9 (6, 16)	>0.9
Unknown	35	1	​	34	2	​	29	7	​	23	13	​
PSA lowest postradical, ng/mL	0.06 (0.03, 0.20)	0.07 (0.05, 0.17)	0.5	0.06 (0.03, 0.17)	0.54 (0.38, 0.70)	0.090	0.06 (0.03, 0.17)	0.08 (0.01, 0.25)	0.7	0.06 (0.03, 0.21)	0.07 (0.03, 0.14)	0.5
Unknown	577	72	​	642	6	​	530	113	​	562	83	​
Family history of radiotherapy toxicity	​	​	>0.9	​	​	>0.9	​	​	0.7	​	​	0.002
0 = No	469 (97%)	56 (98%)	​	518 (97%)	6 (100%)	​	425 (97%)	94 (98%)	​	438 (98%)	84 (91%)	​
1 = Yes	15 (3.1%)	1 (1.8%)	​	16 (3%)	0 (0%)	​	14 (3.2%)	2 (2.1%)	​	8 (1.8%)	8 (8.7%)	​
9 = Not known	0 (0%)	0 (0%)	​	0 (0%)	0 (0%)	​	0 (0%)	0 (0%)	​	0 (0%)	0 (0%)	​
Unknown	289	31	​	318	2	​	255	63	​	280	39	​
Rheumatoid arthritis	​	​	0.3	​	​	>0.9	​	​	0.4	​	​	0.7
1 = Yes	9 (1.2%)	2 (2.3%)	​	11 (1.3%)	0 (0%)	​	8 (1.2%)	3 (1.9%)	​	9 (1.2%)	2 (1.5%)	​
0 = No	764 (99%)	86 (98%)	​	841 (99%)	8 (100%)	​	686 (99%)	156 (98%)	​	717 (99%)	129 (98%)	​
Diabetes	​	​	0.8	​	​	>0.9	​	​	0.9	​	​	0.6
1 = Yes	90 (12%)	11 (13%)	​	100 (12%)	1 (13%)	​	82 (12%)	18 (11%)	​	84 (12%)	17 (13%)	​
0 = No	683 (88%)	77 (88%)	​	752 (88%)	7 (88%)	​	612 (88%)	141 (89%)	​	642 (88%)	114 (87%)	​
Hip replacement	​	​	0.2	​	​	>0.9	​	​	0.043	​	​	0.054
0 = No	753 (97%)	83 (94%)	​	827 (97%)	8 (100%)	​	679 (98%)	150 (94%)	​	709 (98%)	124 (95%)	​
1 = Unilateral	16 (2.1%)	4 (4.5%)	​	20 (2.3%)	0 (0%)	​	12 (1.7%)	8 (5%)	​	15 (2.1%)	5 (3.8%)	​
2 = Bilateral	4 (0.5%)	1 (1.1%)	​	5 (0.6%)	0 (0%)	​	3 (0.4%)	1 (0.6%)	​	2 (0.3%)	2 (1.5%)	​
Radical prostatectomy	​	​	0.011	​	​	0.7	​	​	0.6	​	​	<0.001
0 = No	531 (69%)	72 (82%)	​	597 (70%)	5 (63%)	​	489 (70%)	109 (69%)	​	534 (74%)	65 (50%)	​
1 = Yes	242 (31%)	16 (18%)	​	255 (30%)	3 (38%)	​	205 (30%)	50 (31%)	​	192 (26%)	66 (50%)	​
Angiotensin-converting enzyme inhibitor	​	​	0.12	​	​	0.2	​	​	0.8	​	​	0.7
1 = Yes	190 (25%)	15 (17%)	​	205 (24%)	0 (0%)	​	167 (24%)	37 (23%)	​	171 (24%)	33 (25%)	​
0 = No	583 (75%)	73 (83%)	​	647 (76%)	8 (100%)	​	527 (76%)	122 (77%)	​	555 (76%)	98 (75%)	​
Statins	​	​	0.4	​	​	0.7	​	​	0.2	​	​	0.8
1 = Yes	270 (35%)	35 (40%)	​	303 (36%)	2 (25%)	​	254 (37%)	49 (31%)	​	256 (35%)	48 (37%)	​
0 = No	502 (65%)	53 (60%)	​	548 (64%)	6 (75%)	​	439 (63%)	110 (69%)	​	469 (65%)	83 (63%)	​
Unknown	1	0	​	1	0	​	1	0	​	1	0	​
PDE5 inhibitor	​	​	0.6	​	​	>0.9	​	​	>0.9	​	​	0.7
1 = Yes	11 (1.4%)	0 (0%)	​	11 (1.3%)	0 (0%)	​	9 (1.3%)	2 (1.3%)	​	9 (1.2%)	2 (1.5%)	​
0 = No	760 (99%)	88 (100%)	​	839 (99%)	8 (100%)	​	683 (99%)	157 (99%)	​	715 (99%)	129 (98%)	​
Unknown	2	0	​	2	0	​	2	0	​	2	0	​
Sildenafil	​	​	0.2	​	​	>0.9	​	​	>0.9	​	​	0.3
1 = Yes	18 (2.3%)	0 (0%)	​	18 (2.1%)	0 (0%)	​	15 (2.2%)	3 (1.9%)	​	17 (2.3%)	1 (0.8%)	​
0 = No	755 (98%)	88 (100%)	​	834 (98%)	8 (100%)	​	679 (98%)	156 (98%)	​	709 (98%)	130 (99%)	​
5-α Reductase inhibitor	20 (2.6%)	3 (3.4%)	0.7	23 (2.7%)	0 (0%)	>0.9	20 (2.9%)	3 (1.9%)	0.6	21 (2.9%)	2 (1.5%)	0.6
α-Blocker	​	​	0.003	​	​	>0.9	​	​	0.5	​	​	0.6
1 = Yes	72 (9.3%)	17 (19%)	​	89 (10%)	0 (0%)	​	69 (9.9%)	19 (12%)	​	73 (10%)	15 (11%)	​
0 = No	701 (91%)	71 (81%)	​	763 (90%)	8 (100%)	​	625 (90%)	140 (88%)	​	653 (90%)	116 (89%)	​
Amiodarone	​	​	>0.9	​	​	>0.9	​	​	>0.9	​	​	>0.9
1 = Yes	1 (0.1%)	0 (0%)	​	1 (0.1%)	0 (0%)	​	1 (0.1%)	0 (0%)	​	1 (0.1%)	0 (0%)	​
0 = No	772 (100%)	88 (100%)	​	851 (100%)	8 (100%)	​	693 (100%)	159 (100%)	​	725 (100%)	131 (100%)	​
Hormone therapy	​	​	>0.9	​	​	>0.9	​	​	>0.9	​	​	>0.9
0 = No	233 (30%)	27 (31%)	​	259 (30%)	2 (25%)	​	211 (30%)	48 (30%)	​	220 (30%)	40 (31%)	​
1 = Yes	540 (70%)	61 (69%)	​	593 (70%)	6 (75%)	​	483 (70%)	111 (70%)	​	506 (70%)	91 (69%)	​
Radiotherapy type	​	​	0.014	​	​	0.6	​	​	0.034	​	​	<0.001
3D conformal	248 (32%)	18 (20%)	​	265 (31%)	1 (13%)	​	230 (33%)	36 (23%)	​	236 (33%)	30 (23%)	​
IMRT	167 (22%)	15 (17%)	​	178 (21%)	2 (25%)	​	140 (20%)	39 (25%)	​	135 (19%)	44 (34%)	​
RapidArc/VMAT	358 (46%)	55 (63%)	​	409 (48%)	5 (63%)	​	324 (47%)	84 (53%)	​	355 (49%)	57 (44%)	​
External beam dose, Gy	72 (64, 75)	66 (60, 75)	0.043	70 (60, 75)	75 (72, 76)	0.2	72 (60, 75)	70 (60, 75)	0.6	74 (61, 75)	70 (60, 74)	<0.001
Fractions	35 (25, 37)	29 (20, 37)	0.005	35 (20, 37)	36 (30, 38)	0.6	35 (20, 37)	35 (20, 37)	0.4	35 (24, 37)	35 (20, 37)	<0.001
Pelvic radiation	​	​	0.10	​	​	0.003	​	​	0.2	​	​	>0.9
0 = No	580 (75%)	73 (83%)	​	650 (76%)	2 (25%)	​	533 (77%)	114 (72%)	​	550 (76%)	99 (76%)	​
1 = Yes	193 (25%)	15 (17%)	​	202 (24%)	6 (75%)	​	161 (23%)	45 (28%)	​	176 (24%)	32 (24%)	​
Pelvic dose, Gy	50 (46, 50)	50 (44, 54)	>0.9	50 (46, 50)	45.5 (44, 50)	0.12	50 (46, 50)	50 (46, 50)	0.4	50 (46, 50)	48 (46, 50)	0.3
Unknown	582	73	​	652	2	​	534	115	​	552	99	​
Brachytherapy	​	​	>0.9	​	​	>0.9	​	​	>0.9	​	​	0.8
0 = No	709 (92%)	81 (92%)	​	783 (92%)	8 (100%)	​	639 (92%)	146 (92%)	​	669 (92%)	120 (92%)	​
1 = Yes	64 (8.3%)	7 (8%)	​	69 (8.1%)	0 (0%)	​	55 (7.9%)	13 (8.2%)	​	57 (7.9%)	11 (8.4%)	​
BED_late_	123 (117, 125)	121 (118, 125)	0.6	123 (117, 125)	125 (123, 127)	0.061	123 (117, 125)	121 (117, 125)	0.7	123 (117, 127)	121 (116, 123)	0.003
Mean hours from 12:00 am (solar)	12.08 (9.58, 14.24)	11.92 (9.72, 13.69)	0.5	12.06 (9.59, 14.21)	11.01 (9.28, 12.37)	0.3	12.08 (9.51, 14.29)	11.77 (9.76, 13.81)	>0.9	12 (9.61, 14.24)	12.40 (9.55, 14.21)	0.9
SD of treatment time	1.15 (0.47, 1.84)	1.79 (0.96, 2.30)	<0.001	1.17 (0.49, 1.88)	1.53 (1.10, 2.06)	0.2	1.16 (0.49, 1.86)	1.30 (0.47, 2.05)	0.4	1.15 (0.44, 1.86)	1.45 (0.78, 2.10)	0.002
SD of treatment time	​	​	0.051	​	​	0.3	​	​	0.5	​	​	>0.9
Below 3 hours	742 (96%)	80 (91%)	​	814 (96%)	7 (88%)	​	664 (96%)	150 (94%)	​	694 (96%)	125 (95%)	​
Above 3 hours	31 (4%)	8 (9.1%)	​	38 (4.5%)	1 (13%)	​	30 (4.3%)	9 (5.7%)	​	32 (4.4%)	6 (4.6%)	​
Mean day length/hours	12.81 (9.98, 14.85)	12.47 (10.26, 14.70)	0.7	12.75 (9.99, 14.85)	14.22 (10.56, 15.01)	0.5	12.82 (10.06, 14.76)	12.25 (9.83, 15)	0.5	12.76 (10.02, 14.83)	12.99 (9.93, 15.14)	0.8
Mean solar zenith/degrees	46 (28, 59)	42 (27, 54)	0.074	45 (28, 59)	50 (30, 58)	0.8	46 (28, 59)	41 (27, 58)	0.2	46 (28, 59)	45 (27, 58)	0.5
Mean hours since sunrise	6.30 (3.93, 8.65)	5.69 (4.06, 8.17)	0.5	6.27 (3.94, 8.62)	6.32 (2.37, 8.10)	0.6	6.41 (3.87, 8.70)	5.91 (4.18, 8.09)	0.6	6.27 (3.95, 8.56)	6.43 (3.78, 8.95)	0.8

Abbreviations: IMRT, intensity-modulated radiotherapy; VMAT, volumetric modulated arc therapy.

a Median (Q1, Q3); *n* (%).

b Wilcoxon rank-sum test; Fisher exact test; Pearson χ^2^ test.

### Inclusion/exclusion criteria

Inclusion criteria were men over the age of 18 undergoing external beam radiotherapy (with or without brachytherapy) for prostate cancer, including patients undergoing prior radical prostatectomy with no other malignancy in the past 5 years before treatment (except basal cell or squamous cell carcinoma of the skin). Patients gave informed consent. Exclusion criteria included metastatic disease, prior irradiation at the same site, planned use of protons or high-intensity ultrasound, limited life expectancy, and known HIV infection/infectious hepatitis. Full details of all inclusion and exclusion criteria are provided in ref. 11.

### Endpoints

A deterioration of one or more grades (health professional–scored CTCAE v4.0) from baseline for RB, UI, diarrhea (2+ grades), and UF, as assessed by a healthcare professional at 2 years following radiotherapy.

### Predictors

Putative predictors for toxicity regression models are shown in Table 1 and include patient factors, treatment variables (including medications and radiotherapy), and treatment time variables. Single-nucleotide polymorphisms (SNP) in known circadian genes are detailed in Table 2. To adjust for different radiotherapy regimens, dose was calculated as the biologically effective dose (BED), a widely used method of comparing different radiation treatment fractionation schedules, which assigns a numerical score based on the linear quadratic model. The α value is the number of logs of cell kill per Gray (Gy) from the linear portion of the cell survival curve, and the β value is the number of logs of cell kill per Gy squared from the quadratic component. BED is the product of the number of fractions (*n*); dose per fraction (*d*), including any boost doses; and a factor determined by the dose and α/β ratio [3 Gy for late effects (12)].

$$BED=nd\;\left(1+\frac{d}{\alpha /\beta }\right)$$

**Table 2.** Genes and SNP investigated for interaction with treatment time on radiotherapy side effects.

Gene	rs number	Chr	Position (GRCh38)	MAF^a^ (global)	MAF^a^ (European)	Reason tested
*CLOCK*	rs1801260	4	55,435,202	0.26	0.28	Statistically significant in Webb and colleagues (*P* = 0.02; ref. 6)
*HCRTR2*	rs35833281	6	55,156,763	0.17	0.20	Statistically significant in GWAS for morningness (*P* = 3 × 10^−9^)
*NOCT*	rs13116075	4	139,008,878	0.14	0.16	Validation from Johnson and colleagues (*P* = 5 × 10^−3^; ref. 8) and Webb and colleagues (not significant; ref. 6)
*PER3*	rs172933	1	7,784,620	0.24	0.21	Strong LD with *PER3* VNTR [validation from Johnson and colleagues (*P* = 0.006; ref. 8)]
​	rs2087947	1	7,791,033	0.17	0.2	Validation from Webb and colleagues (*P* < 0.04; ref. 6); strong LD with *PER3* VNTR [validation from Johnson and colleagues (*P* = 0.006; ref. 8)]
​	rs696305	1	7,792,737	0.39	0.35	Strong LD with *PER3* VNTR [validation from Johnson and colleagues (*P* = 0.006; ref. 8)]
​	rs35128310	1	7,800,841	0.48	0.36	Strong LD with *PER3* VNTR [validation from Johnson and colleagues (*P* = 0.006; ref. 8)]
​	rs488728	1	7,801,285	0.42	0.37	Strong LD with *PER3* VNTR [validation from Johnson and colleagues (*P* = 0.006; ref. 8)]
​	rs228640	1	7,802,512	0.27	0.30	Strong LD with *PER3* VNTR [validation from Johnson and colleagues (*P* = 0.006; ref. 8)]
​	rs228644	1	7,806,023	0.38	0.40	Strong LD with *PER3* VNTR [validation from Johnson and colleagues (*P* = 0.006; ref. 8)]
​	rs697689	1	7,823,557	0.20	0.17	Strong LD with *PER3* VNTR [validation from Johnson and colleagues (*P* = 0.006; ref. 8)]
​	rs228697	1	7,827,519	0.10	0.11	Strong LD with *PER3* VNTR [validation from Johnson and colleagues (*P* = 0.006; ref. 8)]
​	rs228661	1	7,832,563	0.15	0.21	Strong LD with *PER3* VNTR [validation from Johnson and colleagues (*P* = 0.006; ref. 8)]
*RASD1*	rs11545787	17	17,494,964	0.22	0.24	Statistically significant in Webb and colleagues (*P* < 0.02; ref. 6). Statistically significant in GWAS for morningness (*P* = 1.4 × 10^−8^)
*VAMP3*	rs11121022	1	7,776,599	0.35	0.40	Strong LD with *PER3* VNTR [validation from Johnson and colleagues (*P* = 0.006; ref. 8)] Statistically significant in GWAS for morningness (*P* = 2 × 10^−8^)

Abbreviation: GWAS, genome-wide association study.

a Source: Allele Frequency Aggregator release 20250407_153717 (May 2025). https://www.ncbi.nlm.nih.gov/snp/docs/gsr/alfa/.

### Treatment times

Local time and date of each radiotherapy fraction were converted into solar time using the photobiology R package (v0.10.5; ref. 13), thus adjusting for longitude, time zone, daylight savings, and seasonal solar orbit variations. Mean treatment time as a continuous predictor was used as a more powerful alternative to grouping by morning/afternoon used in other studies, whereas additional metrics day length, maximum solar elevation, and SD of the fraction times were also calculated. Individual fraction times for each patient varied, so patients with a wide range of treatment times (SD > 3 hours) were excluded, as it is not possible to analyze the contribution of time of day in such patients. As we predict a daily cyclical response to treatment time, we calculated hourly offsets [e.g., 10:00 am and 2:00 pm are both represented as 2 hours from 12:00 pm] to determine the time of lowest/highest toxicity according to model best fits (determined by ROC-AUC).

### Genetic analysis

Genotyping was carried out centrally using Illumina Infinium OncoArrays on 600k SNP and was subjected to standard quality control procedures. A total of 7,409,901 SNP variants were imputed with minor allele frequency (MAF) >0.05 using the 1000 Genome Project data. A power calculation (*n* = 877, power = 0.8, α = 0.01, prevalence = 0.15, relative risk = 1.8; https://doi.org/10.1101/164343 shows sufficient power for 5 independent loci (MAF ≥0.15). SNP were selected in loci in which an SNP * treatment time interaction has been previously investigated (*CLOCK*, *HCRTR2*, *NOCT*, *PER3*/*VAMP3*, *RASD1*; Table 2; ref. 6). As genotyping data for the *PER3* variable number tandem repeat (VNTR; rs57875989) were not available, multiple SNP in linkage disequilibrium (LD) were selected to act as proxies.

### Statistical analysis methods

Variables with >0.5% missing data (MRI T stage, pelvic dose, family history of radiotherapy toxicity) and near-zero variance (rheumatoid arthritis, PDE5 inhibitor, sildenafil, 5-α reductase inhibitor, antimuscarinic, amiodarone, hip replacement, and ethnicity) were removed from the analysis. One patient with no body mass index (BMI) data was removed. Endpoints were not imputed, and patients with no outcome recorded were removed from each endpoint analysis (Fig. 1).

To select features for base models, LASSO regression (binomial, mixture = 1) was performed and tuned using fivefold cross-validation to select λ at which maximum ROC-AUC is achieved. Variables with nonzero coefficients were selected for inclusion in fixed-effects and mixed-effects logistic regressions. When not selected by LASSO regression, BMI and BED_late_ were reintroduced into each regression model due to clinical relevance. Individual SNP were added to base models with treatment time and SNP × treatment time interactions.

To assess model performance, ROC-AUC for models were calculated using the performance R package with fivefold cross-validation. The effect of unmodeled confounding factors between treatment centers (e.g., differences in scoring endpoints) was assessed by estimating the intraclass correlation coefficient (ICC) using the performance package (14, 15) on mixed-effects models (lme4 package) with treatment center as the sole random intercept.

Analyses were carried out in R 4.5.0 (RRID:SCR_001905) with the tidyverse 2.0.0 (RRID:SCR_019186; data handling; ref. 16), tidymodels 1.3.0 (feature selection, cross-validation, modeling, and performance assessment; ref. 17), lme4 1.1-37 (RRID:SCR_015654; mixed-effects models; ref. 18), glmnet 4.1-9 (RRID:SCR_015505; LASSO regression; ref. 19), performance 0.14.0 (model assessment; ref. 15), and ggeffects 2.3.0 (RRID:SCR_022496; model-based predictions; ref. 20) packages. The ggplot2 3.5.2 package (RRID:SCR_014601; ref. 21) was used for graphical outputs. Pearson χ^2^, Fisher exact, or Wilcoxon rank-sum tests were performed for univariable analyses, and the gtsummary 2.2.0 (RRID:SCR_021319) package (22) was used for tabulation of results.

We used the STREGA checklist when writing our report (23, 24).

### Ethics

The REQUITE study was reviewed and approved by the North West—Great Manchester East Ethics Committee (UK, reference 14 NW 0035) and by the local ethics committees of all participating centers. REQUITE was registered at www.controlled-trials.com (ISRCTN98496463). All patients provided written informed consent to participate in the study and for the publication of the data included in this article. The study was conducted in accordance with the Declaration of Helsinki.

### Role of funders

Funders had no role in study design, data collection, data analyses, interpretation, or writing of the report.

## Results

### Cohort description and univariable analysis

Summary statistics on patient characteristics and univariable summary statistics for late (2 years after radiotherapy) endpoints RB, UI, diarrhea (grade 2 or higher), and UF are presented in Table 1 and Supplementary Table S1.

Statistically significant predictors (with ≥5% prevalence) for late toxicity were age (RB, *P =* 0.036), BMI (RB, *P =* 0.002), use of α-blockers (RB, *P =* 0.003), radical prostatectomy (RB, *P =* 0.011; UI, *P <* 0.001), radiotherapy type (intensity-modulated radiotherapy vs. 3D conformal vs. RapidArc/volumetric modulated arc therapy; RB, UF, UI; *P <* 0.03), external beam dosage (total dosage and number of fractions; RB and UI *P <* 0.05), pelvic lymph node irradiation (diarrhea, *P =* 0.003), and BED_late_ (UI, *P =* 0.003). Time of treatment–related variables were statistically nonsignificant in univariable analyses, except for the spread of treatment times (SD of treatment times for all fractions), for RB and UI only. Statistically significant differences in all late toxicities were observed for the treatment centers, underlining the limitations of univariable analyses.

### Late RB

RB (late toxicity—an increase in one or more grades from baseline to 2 years after radiotherapy) was selected for further analysis, as it is a common side effect seen in 10% of the REQUITE prostate cohort with available genotyping, treatment time, and RB data (*n* = 821). Grade 1+ RB was present in 3% of our cohort at baseline (before radiotherapy). Only 1 of those 29 patients showed deterioration in RB at 24 months after radiotherapy, whereas 24 patients showed an improvement. To investigate the effects of interactions between treatment time and circadian genotypes on late toxicity, we constructed logistic multiple regression models following an initial round of feature selection (LASSO regression), selecting from clinical variables, patient demographics, radiotherapy dosimetry, and location-derived time variables [e.g., day length, sun angle at solar zenith (incorporates season and latitude in a single predictor)]. Initial models excluded treatment time and genotype variables—these were added in a second-stage analysis to model any potential circadian effect.

A fixed-effects model (Table 3) for late RB included BMI [odds ratio (OR), 0.91; 95% confidence interval (CI), 0.86–0.97; *P =* 0.003], use of α-blockers (OR, 2.40; 95% CI, 1.28–4.31; *P =* 0.004), and spread of treatment time (SD of treatment times; OR, 1.61; 95% CI, 1.26–2.05; *P* < 0.001). BED_late_ was not statistically significant (*P =* 0.5) but was nonetheless included as the sole radiation dose variable in the base model (ROC-AUC, 0.67; 95% CI, 0.60–0.78). Season- and latitude-related variables were discarded (uninformative) during feature selection, implying no seasonal (day length) effect. Use of α-blockers was nonsignificant (*P =* 0.07) in a mixed-effects model (Supplementary Table S2A), which incorporated treatment center as a random effect. All other significant predictors remained significant (ROC-AUC, 0.71; 95% CI, 0.60–0.80) in the mixed-effects model.

**Table 3.** Logistic regression for deterioration in RB from baseline to 2 years after radiotherapy.

Characteristic	rs11121022 (*VAMP3*)			rs172933 (*PER3*)			rs35128310 (*PER3*)			rs488728 (*PER3*)			rs696305 (*PER3*)			rs697689 (*PER3*)		
	OR	95% CI	*P* value^a^	OR	95% CI	*P* value^a^	OR	95% CI	*P* value^a^	OR	95% CI	*P* value^a^	OR	95% CI	*P* value^a^	OR	95% CI	*P* value^a^
SD of treatment times, hours	1.70	1.26–2.31	**<0.001**	1.66	1.23–2.25	**<0.001**	1.65	1.23–2.24	**0.001**	1.65	1.22–2.23	**0.001**	1.64	1.22–2.23	**0.001**	1.66	1.23–2.26	**<0.001**
BMI, kg/m^2^	0.90	0.84–0.96	**0.001**	0.90	0.85–0.96	**0.002**	0.91	0.85–0.97	**0.003**	0.90	0.85–0.96	**0.002**	0.90	0.84–0.96	**0.001**	0.90	0.84–0.96	**0.001**
BED_late_	1	0.99–1.02	0.7	1	0.99–1.02	0.7	1	0.99–1.02	0.6	1	0.99–1.02	0.7	1	0.99–1.02	0.7	1	0.99–1.02	0.7
α-Blocker	2.41	1.24–4.48	**0.007**	2.64	1.35–4.95	**0.003**	2.46	1.26–4.60	**0.006**	2.46	1.26–4.58	**0.006**	2.34	1.19–4.37	**0.010**	2.62	1.34–4.93	**0.004**
Time from sunrise	1.14	1–1.30	**0.045**	1.24	0.97–1.59	0.088	1.21	0.99–1.49	0.070	1.21	1.01–1.47	**0.043**	0.88	0.78–0.99	**0.038**	0.93	0.85–1.03	0.2
SNP	2.64	1.23–5.67	**0.013** (0.064)	3.04	1.10–9.08	**0.038** (0.2)	2.97	1.19–7.71	**0.022** (0.11)	2.77	1.16–6.82	**0.024** (0.12)	0.34	0.14–0.81	**0.017** (0.087)	0.21	0.06–0.67	**0.012** (0.061)
Time * SNP	0.85	0.76–0.96	**0.008** **(0.04)**	0.87	0.75–1.01	0.064 (0.3)	0.87	0.76–0.99	**0.041** (0.2)	0.86	0.76–0.98	**0.021** (0.1)	1.20	1.06–1.37	**0.005** **(0.027)**	1.23	1.04–1.45	**0.014** (0.07)
AIC	505			507			506			506			503			505		
No. observations	821			821			821			821			821			821		

NOTE: Time origin: sunrise.

a *P* value (adjusted *P* value; false discovery rate corrected based on 5 independent loci). Statistically significant *P* values < 0.05 are shown in bold.

Analysis proceeded using fixed-effects models as intergroup variability is low (ICC rho = 0.09), and such models are both easier to interpret and produce more generalizable predictive models than mixed-effects models. To elucidate the peak time of day for radiotoxicity, we constructed multiple models, whereby treatment time was fitted as the number of hours away from different “origin” times of the day (e.g., from a 12:00 pm reference time point, both 9:00 am and 3:00 pm treatment times are treated as +3 hours). In addition, an interaction term between time and circadian genotype allowed any circadian effects to be modeled while taking genotype into consideration. Comparing the overall performance (ROC-AUC) of each treatment time origin for each SNP in combination showed no improvement in model performance over the base model. Mean ROC-AUC per time origin for all SNP models was 0.64 to 0.65, with the highest for time from sunrise (ROC-AUC, 0.65; 95% CI, 0.57–0.73). Treatment time in these interaction models was statistically significant for some SNP (rs11121022, rs488728, rs696305; OR, 0.88–1.21; *P* < 0.05), but not others (OR, 0.93–1.21; *P =* 0.07–0.2).

**Figure 2.**
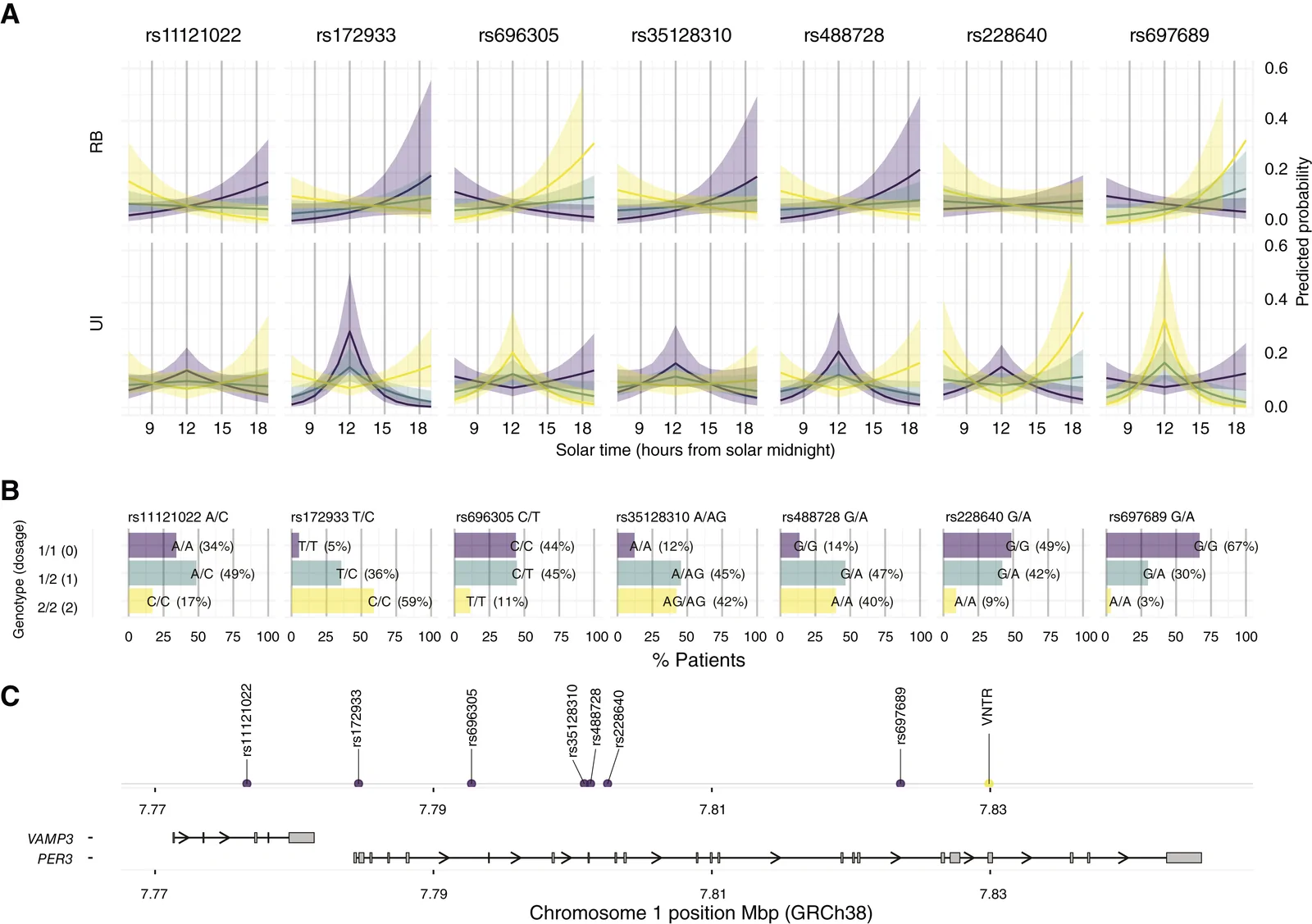
**A,** Predicted probability (*y*-axis) of a patient developing late RB (top row) and late UI (second row) as derived from fixed-effects regression models for different treatment times (solar time of day, *x*-axis) and genotype for *VAMP3* SNP rs11121022 and adjacent *PER3* SNP rs172933, rs696305, rs35128310, rs488728, rs288640, and rs697689. Probabilities of developing each late side effect are given for patient groups grouped by genotype at each SNP. Possible patient genotypes are colored as follows: purple, homozygous for allele 1; green, heterozygous; yellow, homozygous for allele 2. Alleles are arbitrarily assigned as allele 1 or allele 2, and their assignments can be determined from **B**. Shaded areas show 95% CI for predictions. **B,** Proportion of patients in the study cohort (European population) with given genotypes for each SNP. Colors represent genotypes for homozygous alleles 1/1 (purple), heterozygotes (green), and homozygous allele 2 (yellow). Actual SNP alleles are provided in the text labels along with the expected proportion of patients with those genotypes according to population frequency estimates. **C,** Chromosome diagram showing the position of each SNP on chromosome 1 (GRCh38). The position of *PER3* VNTR rs57875989 is shown in exon 18. Gene structures of *VAMP3* and *PER3* are shown beneath.

Five statistically significant SNP for RB were found in *PER3* [rs172933 (OR, 3; 95% CI, 1.1–9.1; *P =* 0.038), rs35128310 (OR, 3; 95% CI, 1.2–7.7; *P =* 0.022), rs488728 (OR, 2.8; 95% CI, 1.2–6.8; *P =* 0.024), rs696305 (OR, 0.34; 95% CI, 0.14–0.81; *P =* 0.017), and rs697689 (OR, 0.21; 95% CI, 0.06–0.67; *P =* 0.012)] and one 8 kb upstream of the closest *PER3* SNP in the neighboring gene *VAMP3* [rs11121022 (OR, 2.6; 95% CI, 1.2–5.7; *P =* 0.013; Table 3)]. In addition, we noted a significant interaction exists between all significant SNP and treatment time (*P =* 0.005–0.041), except rs172933 (nonsignificant *P =* 0.064), suggesting that circadian genotypes modify the time of peak radiotoxicity risk. No individual SNP remained statistically significant after correction for multiple testing [false discovery rate (FDR)], but the interactions of rs696305 and rs11121022 with treatment time remain statistically significant (*P =* 0.027 and *P =* 0.04). Although this circadian effect is statistically significant, the effect remains small. Given the low ORs of treatment time and the gene–time interaction, this translates to only a small modification of the risk of RB within normal daily hospital treatment windows. Model-based predictions for a typical patient showed that 44% of patients (with rs696305 C/C) could potentially reduce their risk of RB from 11% when treated at 9:00 am to 5% when treated at 5:00 pm (Fig. 2).

Late UI (a change of 1+ grades from baseline at 2 years after radiotherapy) was seen in 15% of patients (*n* = 857). Following feature selection, our base model (logistic regression; fixed-effects) included radical prostatectomy (OR, 3.3; 95% CI, 2.2–4.9; *P* < 0.0001) and spread of treatment times (SD of treatment times for all fractions; OR, 1.61; 95% CI, 1.25–2.07; *P =* 0.0002). BMI and BED_late_ were also included in the base model on the basis of clinical relevance, despite not being statistically significant. The fixed model had an ROC-AUC of 0.69 (95% CI, 0.6–0.77), whereas a mixed-effects model using treatment center as a random intercept had an ROC-AUC of 0.71 (95% CI, 0.62–0.76; Supplementary Table S2B). Radical prostatectomy and spread of treatment times remain statistically significant, and BMI and BED_late_ remained statistically nonsignificant. Low ICC = 0.06 suggests that most intersite variability is captured by the fixed-effects model.

Constructing multiple models for different times of treatment offsets showed that the best-fitting models (Table 4) are based on time from 12:00 pm, with a mean ROC-AUC of 0.68 (95% CI, 0.63–0.73) across all considered SNP. Treatment time (from solar 12:00 pm) is significant [OR, 0.52 (95% CI, 0.35–0.77); *P =* 0.002], indicating an overall peak in toxicity around midday, decreasing earlier into the morning and later into the afternoon. Five SNP, all in *PER3* and encompassing 39 kb, were found to be statistically significant: rs172933 (OR, 0.44; 95% CI, 0.24–0.82; *P =* 0.008), rs228640 (OR, 0.50; 95% CI, 0.26–0.93; *P =* 0.032), rs488728 (OR, 0.52; 95% CI, 0.30–0.90; *P =* 0.019), rs696305 (OR, 1.8; 95% CI, 1.0–3.1; *P =* 0.033), and rs697689 (OR, 2.4; 95% CI, 1.3–4.7; *P =* 0.008). rs172933 and rs697689 remain statistically significant following FDR correction (5 loci, *P <* 0.05). Statistically significant interactions between genotype and time of treatment were present for all 5 SNP (*P <* 0.01, FDR corrected *P* ≤ 0.05).

**Table 4.** Logistic regression for deterioration in UI from baseline to 2 years after radiotherapy.

Characteristic	rs172933 (*PER3*)			rs228640 (*PER3*)			rs488728 (*PER3*)			rs696305 (*PER3*)			rs697689 (*PER3*)		
	OR	95% CI	*P* value (q value)	OR	95% CI	*P* value (q value)	OR	95% CI	*P* value (q value)	OR	95% CI	*P* value (q value)	OR	95% CI	*P* value (q value)
Radical prostatectomy	3.34	2.22–5.05	**<0.001**	3.43	2.28–5.18	**<0.001**	3.40	2.26–5.13	**<0.001**	3.44	2.29–5.20	**<0.001**	3.41	2.27–5.16	**<0.001**
SD treatment time (solar)	1.66	1.28–2.16	**<0.001**	1.61	1.25–2.09	**<0.001**	1.60	1.24–2.08	**<0.001**	1.59	1.23–2.06	**<0.001**	1.64	1.27–2.13	**<0.001**
BMI	1.03	0.98–1.07	0.2	1.03	0.98–1.07	0.3	1.03	0.98–1.07	0.2	1.03	0.98–1.07	0.2	1.03	0.98–1.08	0.2
BED_late_	0.99	0.98–1	0.11	0.99	0.98–1	0.14	0.99	0.98–1	0.13	0.99	0.98–1	0.2	0.99	0.98–1	0.12
Time (hours)	0.49	0.32–0.73	**<0.001**	0.77	0.64–0.94	**0.010**	0.63	0.46–0.86	**0.003**	1.11	0.92–1.33	0.3	1.08	0.92–1.27	0.3
SNP	0.44	0.24–0.82	**0.008** **(0.04)**	0.50	0.26–0.93	**0.032** (0.16)	0.52	0.30–0.90	**0.019** (0.1)	1.81	1.04–3.12	**0.033** (0.16)	2.44	1.26–4.69	**0.008** **(0.04)**
Time * SNP	1.52	1.20–1.95	**<0.001** **(0.003)**	1.36	1.10–1.69	**0.005** **(0.03)**	1.35	1.10–1.66	**0.004** **(0.02)**	0.76	0.62–0.94	**0.010** **(0.05)**	0.66	0.51–0.85	**0.002** **(0.009)**
AIC	651	​	​	655	​	​	655	​	​	657	​	​	653	​	​
No. observations	818	​	​	818	​	​	818	​	​	818	​	​	818	​	​

NOTE: Time origin: 12:00 pm. q value: FDR corrected *P* value, based on 5 independent loci. Statistically significant *P* and q values (< 0.05) are shown in bold.

The best-fitting fixed-effects model (rs172933 * time from 12:00 pm) had an ROC-AUC of 0.71 (95% CI, 0.63–0.82) and predicted a significant reduction in the risk of UI from 15% (95% CI, 10%–23%) to 5% (95% CI, 2%–8%) by avoiding treatment in the middle of the day for patients heterozygous for rs172933 (T/C; 36% of patients). Conversely, scheduling treatment for the middle of the day could provide a smaller benefit for 59% of patients (rs172933 C/C genotype), reducing the risk of UI from 13% (95% CI, 8%–20%) in the early morning and late evening to 7% (95% CI, 4%–12%) around noon.

### Interim time points and late effects beyond 2 years

Data on side effects at 12 months after radiotherapy were available for most patients, with toxicity data at 24 months [RB, *n* = 799 (97%); UI, *n* = 795 (97%)]. As very limited data were collected at the 36-month visits with a substantial loss to follow-up (*n* = 303 remaining; due to the 2020 COVID-19 pandemic), we did not analyze these late time points as they would likely introduce substantial bias.

Many patients develop side effects by 12 months who then improve before the 24-month visit; conversely, many patients with no deterioration from baseline at 12 months go on to develop side effects by 24 months. For RB and UI, we carried out 2 additional sensitivity analyses, reconstructing our fixed-effects models with (i) the removal of patients with a deterioration from baseline at 12 months who then improve to show no deterioration from baseline at 24 months [RB, *n* = 36 (50% of patients improve), UI, *n* = 47 (45% improve)] and (ii) grouping patients with a deterioration from baseline at either 12 or 24 months after radiotherapy (RB *n* = 116; UI *n* = 171). For RB, SNP × time interactions remain significant for rs11121022, rs488728, rs696305, and rs697689 for analysis 1, but only rs11121022 and rs696305 remained significant for analysis 2 (Supplementary Table S3A and S3B). For UI, all SNP × time interactions remained statistically significant for both analyses (Supplementary Table S4A and S4B).

### Other endpoints

Diarrhea and UF were analyzed with the same methods as RB and late UI above but did not reveal any statistically significant SNP or interactions with treatment time.

## Discussion

We showed previously in patients with breast cancer that the time of day at which radiotherapy fractions are delivered can affect their risk of developing both acute and late (2+ years) side effects. Daily timing of radiotherapy side effect risk maxima and minima was modified according to variants in circadian clock genes, with a modification of the timing of cell-cycle phases (25)—and thus the corresponding timing of peak radiosensitivity phases (highest at the G_2_–M transition and M-phases)—postulated as a possible explanation. In turning our attention to prostate cancer, applying the same analysis methods and candidate gene selections, we now show the same effect is seen in nonbreast tissues and in a different cancer type.

Our initial univariable analyses revealed few surprises. Risk factors such as age and BMI and treatment factors such as preradiotherapy surgery, radiation dose, and modality all modify risk across one or more of our late toxicity endpoints. Increased BMI was protective for RB (OR, 0.9; 95% CI, 0.85–0.96; *P =* 0.003), as we would expect: Increased space between the prostate and rectal wall effectively reduces the radiologic dose delivered to the rectum. The use of α-blockers—routinely used to increase urinary flow in patients with benign prostatic hypertrophy, as well as prostate cancer—is a risk factor for late RB in both uni- and multivariable analyses. RB varied between our treatment centers (6.6%–29%), and in mixed-effect models—which account for recruitment center—α-blocker use was statistically nonsignificant (*P =* 0.07). Some of the relationship between α-blocker use and late RB may instead be accounted for by other intercenter variations (e.g., differences in symptom scoring) not captured in our fixed-effects models (BMI, radiation dose, and spread of treatment times).

Prior radical prostatectomy increased late UI (OR, 3.28; 95% CI, 2.20–4.90; *P* < 0.0001). A higher prevalence of late UI following radical prostatectomy was also reported in the PACE-A trial (26).

Treatment time considered alone was nonsignificant for late radiotoxicities in univariable models and also nonsignificant in multivariable logistic regression models for late RB and late UI. This fits with mixed findings in the literature on treatment timing and radiotherapy effects (5, 7, 8, 27–30). Treatment time was only significant once interactions with circadian genotypes were introduced into multivariable models, implying that opposing treatment time effects are present in subgroups of patients defined by genotypes in circadian genes. When all subgroups are considered together, the overall effect is largely canceled out, with the largest subgroup driving the diminished overall effect.

Our previous study in breast cancer identified SNP in circadian genes *CLOCK*, *RASD1*, and *PER3* (6), whereas the present study only identified SNP in *PER3.* The breast cancer study not only had a larger sample size but was also better powered owing to the higher prevalence of late breast atrophy (∼30%) compared with RB and late UI here (10% and 15%, respectively). For both RB and UI, multiple SNP within the *PER3* gene interact with treatment time and demonstrate a circadian effect. All statistically significant *PER3* markers show strong LD with each other (|D′| = 0.63–1) and thus should not be considered independent effects. These *PER3* SNP were selected due to their strong association with a 4- or 5-repeat 54 bp coding region VNTR. This VNTR lies within a coding region of *PER3* (exon 18), with each repeat motif encoding 18 amino acids. Modifying the structure of the PER3 protein influences its interaction with other proteins through tertiary structure modification and phosphorylation, including core clock proteins. This VNTR has been previously linked to delayed sleep phase syndrome as well as diurnal preference (determined by sleep questionnaires), with *PER3* 5/5 conferring “morningness” and *PER3* 4/4 conferring “eveningness” (31). In contrast, more recent articles found no significant relationship between VNTR repeat length alone and morningness/eveningness (32, 33), but one describes an effect in which the G allele of exon 17 SNP rs228697 confers morningness, and that association is strengthened further when combined with the 4-repeat version of the *PER3* VNTR. In addition, 4/4 types showed advanced sleep–wake time when morning light was administered (33).

Although *PER3* is primarily expressed during the night/early morning, translational lag will delay the build-up of PER3 protein within the cytoplasm. PER3 influences the peripheral clock by forming heterodimers with PER1/2 and CRY1/2 before entering the nucleus and inhibiting the expression of *CLOCK*/*BMAL1* (34). The circadian effect of *PER3* depends not only on its own transcription but also on the rhythmic expression of other circadian clock components such as PER1/2 and CRY1/2. *PER3* knockout human bone osteosarcoma epithelial cells have demonstrated that PER3 is not essential to the maintenance of human circadian rhythms but instead modifies period length (rather than shifts the phase). Matsumura and colleagues (35) postulate that the circadian period is elongated as PER3-CRY1/2 heterodimers translocate to the nucleus more slowly than other PER–CRY complexes. The *PER3* SNP—with the notable exception of rs228697—in our study are noncoding (intronic or 5′ untranslated region) and have no known functional phenotype. They were selected on the basis of being in strong LD with the *PER3* VNTR, and we expect that any effect seen is due to their association with the VNTR. The additional 18 amino acids in the 5-repeat compared with 4-repeat version of the VNTR present an additional phosphorylation site for CKIε, which affects PER3 stability and transport to the nucleus (31).

rs228697 (alleles C/G) is a nonsynonymous SNP located in exon 17 and results in a P864A amino acid substitution. This substitution is postulated to disrupt the PER3 secondary structure, affecting its accumulation in the cytoplasm and subsequent transfer to the nucleus (33). In a previous study, VNTR 4/4 individuals have only the associated with a morningness phenotype when the rs228697(G) allele has also been present (33). That SNP shows very strong LD with the VNTR (|D′| = 0.91) such that it effectively subdivides the 4-repeat VNTR haplotype into 2. In our study, we find no statistically significant (*P* < 0.05) effect of this SNP [and by extension the rs228697(G)-VNTR(4) subhaplotype], nor any statistically significant interaction with treatment time. This may be a consequence of the SNP’s low MAF (0.10), restricting power and does not necessarily demonstrate the absence of an underlying effect.

The exact mechanism by which *PER3* modifies radiotherapy side effect risk at different times of the day is not known, but *PER3* does play a role in regulating the central clock and peripheral endogenous cellular clocks. These clocks affect a variety of mechanisms that could affect the response of normal tissues to radiotherapy. For example, cell division in proliferating tissues such as the oral and rectal mucosa has been suggested to be tied to the circadian rhythm, with mitosis occurring at night and DNA synthesis in the morning (36, 37). Cells vary in radiosensitivity according to the phase of the cell cycle due to differences in DNA double-strand break repair pathways. PER3 is required for the activation of the ATM/Chk2 DNA damage response pathways (38). Failure to repair DNA in normal tissue leads to cell death or senescence, which may contribute to late side effects caused by chronic inflammation and fibrosis affected by the senescence-associated secretory phenotype (39). In summary, variants in *PER3* affect the peripheral clock due—at least in part—to differences in accumulation in the cytoplasm and translocation to the nucleus, in which it forms part of the feedback loop regulating the expression of *CLOCK*/*BMAL1*. In the cytoplasm, PER3 is involved in triggering the DNA damage response, the failure of which leads to cell death and senescence, resulting in both acute and late side effects from radiotherapy.

Other potential mechanisms include daily rhythms in cytokines known to be important in the development of radiotherapy side effects, for example, TGFβ and TNF (40, 41).

Our models from breast (acute erythema and late atrophy) and prostate (late RB and UI) suggest that different tissues exhibit different peak times of radiosensitivity. This is consistent with findings from a landmark study in the diurnal primate *Papio anubis* (wild olive baboon), in which a comprehensive multiorgan transcription atlas was constructed from circadian-entrained individuals at different times of the day (42). The atlas revealed tissue-specific timings of expression of DNA repair genes despite coordinated circadian clocks, with the implication that certain cell types will be in the most radiosensitive stages of the cell cycle (G_2_–M), whereas others are not.

Tissue-specific timing implies that in some cases, it may be necessary to balance decreasing the risk of one side effect against increasing the risk of another side effect (Fig. 2). Planning the optimum time for radiotherapy should therefore seek to mitigate the overall side effect burden, with priority given to reducing those side effects with the greatest impact on quality of life.

We note that the present study has certain limitations. A larger sample cohort would provide more power to detect further circadian genes as well as SNP with lower MAF, which interact with treatment time. Power considerations mean we have only examined a small number of genetic loci; a significantly larger sample would enable a high-density whole-genome approach. Day length is affected by both season and latitude, and—although we collected data throughout the year and did not note any seasonal effect—we only collected from a narrow range of temperate latitudes. Recruitment from more equatorial regions would allow comparison between areas where day length fluctuates widely and those where day length is more consistent throughout the year. Finally, we also examined a narrow range of dose and fractionation regimens. It is not clear how or if our findings could be extrapolated to ultra-hypofractionated regimens (e.g., up to 5 fractions delivered during 1 working week), but we would expect the overall treatment time and gene interaction effect to remain and the SD of treatment times to be reduced. Depending on scheduling, this may substantially reduce (all fractions delivered away from high-risk periods) or exacerbate (some or all fractions delivered in high-risk periods) the occurrence of side effects, making the findings of this study especially pertinent. Our expectation is that although normal tissue cell cycles remain under circadian control, tumor tissue cells are characteristically heterogeneous, and cell cycles are dysregulated. In such circumstances, the therapeutic window for treating cancer by radiotherapy can be widened with careful planning of treatment times. This effect has been exemplified in a recent study of 488 patients with esophageal squamous cell carcinoma receiving radiotherapy in the daytime (before 7:00 pm) versus in the evening (after 7:00 pm), showing an increased incidence of radiation esophagitis in the evening treatment group but no significant difference in overall survival (43).

Despite limitations, this present study supports the findings of our previous study in breast cancer, which described variants in *PER3* (as well as *CLOCK* and *RASD1*) that interact with the time of treatment to modulate the occurrence of side effects from radiotherapy. We confirm that this circadian effect influences outcomes from treatment not only in different cancer types but also in different tissues. The same chronomodulation effect may be seen in further cancer types, such as head and neck cancer, in which the side effects of radiotherapy have profound quality-of-life implications. The effects already reported in both breast and prostate cancer should now be explored further, including through small validation cohorts and pilot trials targeting genetically guided treatment timing to reduce common radiation side effects.

## Supplementary Material

Supplementary Table S1 Supplementary table 1: Cohort characteristics by treatment centre (site)

Supplementary Table S2 Rectal bleeding and urinary incontinence base models

Supplementary Table S3 Rectal bleeding sensitivity analyses

Supplementary Table S4 Urinary incontinence sensitivity analyses

Supplementary Appendix S1 List of REQUITE Consortium members. Author list on main manuscript refers to this as 'REQUITE Consortium'.

## Data Availability

Funding for the 5-year REQUITE project ended on September 30, 2018. REQUITE does not benefit financially from supplying data and/or samples to researchers but does make a charge to cover its costs and support continued maintenance of the database and biobank beyond the end of the funding period. To facilitate this continued access for researchers, the REQUITE steering committee approved a tiered cost recovery model for access to data and/or samples. Contact REQUITE (requite@le.ac.uk) for more information on pricing and to make an application. Applicants will be asked to complete a concept form, which is then considered by the REQUITE legacy committee. Following a successful application for data access to the REQUITE legacy committee, data are provided via a secure server.
