# Supplementary material for: Circadian Genotypes in PER3 and Time of Prostate Cancer Radiotherapy Interact to Affect Risk of Late Side Effects
Source: Clin Cancer Res. 2026 Jul 13;32(19):4384–95. doi: 10.1158/1078-0432.CCR-26-0268 (PMC13628092; doi:10.1158/1078-0432.CCR-26-0268)
Supplement: Supplementary file 1 [file ccr-26-0268_supplementary_table_s1_suppts1.pdf]

Supplementary table 1: Cohort characteristics by treatment centre (site)

	Overall	Recruitment site					
Characteristic	N = 862[1]	Gent N = 170[1]	Mannheim N = 50[1]	Milan N = 121[1]	Santiago N = 265[1]	Manchester N = 165[1]	Leicester N = 91[1]
Height / cm	173 (167, 178)	175 (170, 179)	177 (173, 182)	175 (170, 178)	166 (162, 170)	176 (172, 180)	176 (170, 183)
Weight / kg	81 (74, 90)	81 (73, 90)	84 (77, 92)	79 (74, 87)	80 (72, 88)	83 (75, 92)	86 (76, 94)
Unknown	1	0	0	0	0	1	0
BMI kg / m^2	27.1 (24.9, 29.8)	26.2 (24.4, 29.2)	27.1 (25.1, 29.6)	26.0 (24.3, 28.3)	28.7 (26.1, 31.2)	26.8 (24.4, 29.4)	27.2 (25.2, 29.7)
Unknown	1	0	0	0	0	1	0
Age at radiotherapy start	70 (65, 74)	69 (63, 74)	71 (63, 74)	70 (64, 74)	71 (66, 75)	71 (67, 75)	68 (64, 72)
Smoker							
0=Never	341 (40%)	51 (30%)	14 (28%)	38 (31%)	125 (47%)	69 (42%)	44 (48%)
1=Ex before cancer diagnosis	399 (46%)	92 (54%)	30 (60%)	64 (53%)	99 (37%)	81 (49%)	33 (36%)
2=Ex since cancer diagnosis	25 (2.9%)	4 (2.4%)	1 (2.0%)	1 (0.8%)	9 (3.4%)	6 (3.6%)	4 (4.4%)
3=Current	96 (11%)	22 (13%)	5 (10%)	18 (15%)	32 (12%)	9 (5.5%)	10 (11%)
7=Do not wish to answer	0 (0%)	0 (0%)	0 (0%)	0 (0%)	0 (0%)	0 (0%)	0 (0%)
Unknown	1	1	0	0	0	0	0
Ethnicity							
1=White (European or American European)	853 (99%)	169 (99%)	50 (100%)	120 (99%)	261 (98%)	163 (100%)	90 (100%)
Other	6 (0.7%)	1 (0.6%)	0 (0%)	1 (0.8%)	4 (1.5%)	0 (0%)	0 (0%)
Unknown	3	0	0	0	0	2	1
MRI T-stage							
T2a	62 (18%)	6 (21%)	1 (10%)	11 (37%)	10 (10%)	13 (15%)	21 (24%)
T2b	35 (10%)	4 (14%)	0 (0%)	0 (0%)	19 (19%)	3 (3.4%)	9 (10%)
T2c	58 (17%)	9 (32%)	2 (20%)	0 (0%)	18 (18%)	16 (18%)	13 (15%)
T3a	95 (28%)	7 (25%)	0 (0%)	6 (20%)	33 (34%)	32 (36%)	17 (19%)
T3b	64 (19%)	2 (7.1%)	0 (0%)	6 (20%)	15 (15%)	20 (22%)	21 (24%)
T4	4 (1.2%)	0 (0%)	1 (10%)	0 (0%)	1 (1.0%)	1 (1.1%)	1 (1.1%)
Not visible	25 (7.3%)	0 (0%)	6 (60%)	7 (23%)	2 (2.0%)	4 (4.5%)	6 (6.8%)
Unknown	519	142	40	91	167	76	3
cN stage							
0=N0	667 (77%)	158 (93%)	25 (50%)	91 (75%)	146 (55%)	163 (99%)	84 (92%)
1=N1	30 (3.5%)	9 (5.3%)	1 (2.0%)	0 (0%)	15 (5.7%)	0 (0%)	5 (5.5%)
2=NX	165 (19%)	3 (1.8%)	24 (48%)	30 (25%)	104 (39%)	2 (1.2%)	2 (2.2%)
pN stage							
0=N0	141 (36%)	62 (60%)	24 (65%)	28 (45%)	15 (13%)	2 (40%)	10 (14%)
1=N1	45 (11%)	34 (33%)	5 (14%)	3 (4.8%)	3 (2.6%)	0 (0%)	0 (0%)
2=NX	206 (53%)	8 (7.7%)	8 (22%)	31 (50%)	97 (84%)	3 (60%)	59 (86%)
Unknown	470	66	13	59	150	160	22
Gleason score (biopsy)							
2	1 (0.1%)	0 (0%)	0 (0%)	0 (0%)	1 (0.4%)	0 (0%)	0 (0%)
4	2 (0.2%)	0 (0%)	0 (0%)	0 (0%)	2 (0.8%)	0 (0%)	0 (0%)
5	9 (1.0%)	2 (1.2%)	0 (0%)	1 (0.8%)	5 (1.9%)	0 (0%)	1 (1.1%)
6	151 (18%)	28 (17%)	10 (20%)	16 (13%)	84 (32%)	11 (6.7%)	2 (2.2%)
7	486 (57%)	85 (50%)	32 (64%)	61 (50%)	134 (51%)	107 (65%)	67 (74%)
8	114 (13%)	26 (15%)	3 (6.0%)	27 (22%)	29 (11%)	27 (16%)	2 (2.2%)
9	94 (11%)	27 (16%)	5 (10%)	15 (12%)	9 (3.4%)	20 (12%)	18 (20%)
10	3 (0.3%)	1 (0.6%)	0 (0%)	1 (0.8%)	1 (0.4%)	0 (0%)	0 (0%)
Unknown	2	1	0	0	0	0	1
PSA pre-diagnostic biopsy / ng/ml	9 (6, 16)	10 (7, 20)	10 (8, 14)	7 (5, 11)	8 (6, 13)	11 (7, 21)	14 (8, 24)
Unknown	36	25	1	2	2	4	2
Family history of radiotherapy toxicity							
0=No	526 (97%)	142 (95%)	24 (96%)	23 (92%)	108 (98%)	140 (99%)	89 (99%)
1=Yes	16 (3.0%)	8 (5.3%)	1 (4.0%)	2 (8.0%)	2 (1.8%)	2 (1.4%)	1 (1.1%)
Unknown	320	20	25	96	155	23	1
Rheumatoid arthritis							
1=Yes	11 (1.3%)	5 (2.9%)	2 (4.0%)	1 (0.8%)	1 (0.4%)	1 (0.6%)	1 (1.1%)
0=No	851 (99%)	165 (97%)	48 (96%)	120 (99%)	264 (100%)	164 (99%)	90 (99%)
Diabetes							
1=Yes	101 (12%)	17 (10%)	6 (12%)	15 (12%)	46 (17%)	6 (3.6%)	11 (12%)
0=No	761 (88%)	153 (90%)	44 (88%)	106 (88%)	219 (83%)	159 (96%)	80 (88%)
Hip replacement							
0=No	837 (97%)	161 (95%)	48 (96%)	120 (99%)	263 (99%)	155 (94%)	90 (99%)
1=Unilateral	20 (2.3%)	8 (4.7%)	2 (4.0%)	1 (0.8%)	2 (0.8%)	6 (3.6%)	1 (1.1%)
2=Bilateral	5 (0.6%)	1 (0.6%)	0 (0%)	0 (0%)	0 (0%)	4 (2.4%)	0 (0%)
Radical prostatectomy							
0=No	604 (70%)	85 (50%)	22 (44%)	88 (73%)	173 (65%)	153 (93%)	83 (91%)
1=Yes	258 (30%)	85 (50%)	28 (56%)	33 (27%)	92 (35%)	12 (7.3%)	8 (8.8%)
ACE inhibitor							
1=Yes	205 (24%)	27 (16%)	6 (12%)	26 (21%)	92 (35%)	33 (20%)	21 (23%)
0=No	657 (76%)	143 (84%)	44 (88%)	95 (79%)	173 (65%)	132 (80%)	70 (77%)
Statins							
1=Yes	306 (36%)	64 (38%)	9 (18%)	21 (17%)	106 (40%)	72 (44%)	34 (37%)
0=No	555 (64%)	106 (62%)	40 (82%)	100 (83%)	159 (60%)	93 (56%)	57 (63%)
Unknown	1	0	1	0	0	0	0
PDE5 inhibitor							
1=Yes	11 (1.3%)	3 (1.8%)	0 (0%)	2 (1.7%)	2 (0.8%)	3 (1.8%)	1 (1.1%)
0=No	849 (99%)	167 (98%)	48 (100%)	119 (98%)	263 (99%)	162 (98%)	90 (99%)
Unknown	2	0	2	0	0	0	0
Sildenafil							
1=Yes	18 (2.1%)	1 (0.6%)	3 (6.0%)	8 (6.6%)	0 (0%)	3 (1.8%)	3 (3.3%)
0=No	844 (98%)	169 (99%)	47 (94%)	113 (93%)	265 (100%)	162 (98%)	88 (97%)
5-alpha reductase inhibitor	23 (2.7%)	1 (0.6%)	1 (2.0%)	0 (0%)	14 (5.3%)	6 (3.6%)	1 (1.1%)
Alpha blocker							
1=Yes	89 (10%)	7 (4.1%)	3 (6.0%)	1 (0.8%)	40 (15%)	34 (21%)	4 (4.4%)
0=No	773 (90%)	163 (96%)	47 (94%)	120 (99%)	225 (85%)	131 (79%)	87 (96%)
Amiodarone							
1=Yes	1 (0.1%)	0 (0%)	1 (2.0%)	0 (0%)	0 (0%)	0 (0%)	0 (0%)
0=No	861 (100%)	170 (100%)	49 (98%)	121 (100%)	265 (100%)	165 (100%)	91 (100%)
Hormone therapy							
0=No	261 (30%)	24 (14%)	46 (92%)	28 (23%)	122 (46%)	32 (19%)	9 (9.9%)
1=Yes	601 (70%)	146 (86%)	4 (8.0%)	93 (77%)	143 (54%)	133 (81%)	82 (90%)
Radiotherapy type							
3D conformal	266 (31%)	1 (0.6%)	0 (0%)	0 (0%)	265 (100%)	0 (0%)	0 (0%)
IMRT	182 (21%)	129 (76%)	0 (0%)	0 (0%)	0 (0%)	53 (32%)	0 (0%)
Rapid ARC / VMAT	414 (48%)	40 (24%)	50 (100%)	121 (100%)	0 (0%)	112 (68%)	91 (100%)

	Overall	Recruitment site					
Characteristic	N = 862[1]	Gent N = 170[1]	Mannheim N = 50[1]	Milan N = 121[1]	Santiago N = 265[1]	Manchester N = 165[1]	Leicester N = 91[1]
External beam dose / Gy	71 (60, 75)	69 (65, 74)	75 (75, 75)	78 (70, 78)	74 (66, 76)	60 (52, 60)	74 (74, 74)
Fractions	35 (20, 37)	25 (25, 37)	35 (35, 35)	39 (35, 39)	37 (33, 38)	20 (20, 20)	37 (37, 37)
Pelvic radiation							
0=No	654 (76%)	132 (78%)	39 (78%)	42 (35%)	224 (85%)	165 (100%)	52 (57%)
1=Yes	208 (24%)	38 (22%)	11 (22%)	79 (65%)	41 (15%)	0 (0%)	39 (43%)
Pelvic dose / Gy	50.0 (46.0, 50.0)	46.9 (45.0, 48.0)	44.0 (44.0, 50.0)	50.0 (50.0, 50.0)	46.0 (46.0, 46.0)	NA (NA, NA)	56.0 (54.0, 56.0)
Unknown	656	134	39	42	224	165	52
Brachytherapy							
0=No	791 (92%)	170 (100%)	50 (100%)	119 (98%)	235 (89%)	126 (76%)	91 (100%)
1=Yes	71 (8.2%)	0 (0%)	0 (0%)	2 (1.7%)	30 (11%)	39 (24%)	0 (0%)
BEDlate	123 (117, 125)	121 (120, 123)	125 (125, 125)	130 (117, 130)	123 (110, 127)	119 (97, 121)	123 (123, 123)
Mean hours from 00:00 (solar)	12.03 (9.60, 14.21)	10.36 (8.89, 12.87)	9.48 (8.37, 10.55)	11.37 (9.39, 14.11)	14.47 (12.38, 15.98)	12.09 (10.17, 13.37)	10.89 (9.58, 12.80)
Standard deviation of treatment time	1.18 (0.49, 1.89)	1.43 (1.00, 1.97)	1.92 (1.21, 2.38)	0.05 (0.04, 0.20)	0.83 (0.41, 1.33)	1.93 (1.30, 2.42)	1.64 (1.34, 2.13)
Standard deviation of treatment time							
Below 3 hrs	823 (95%)	165 (97%)	46 (92%)	121 (100%)	261 (98%)	144 (87%)	86 (95%)
Above 3 hrs	39 (4.5%)	5 (2.9%)	4 (8.0%)	0 (0%)	4 (1.5%)	21 (13%)	5 (5.5%)
Mean day length / hours	12.76 (10.01, 14.85)	12.94 (9.73, 15.46)	12.08 (9.45, 14.72)	13.19 (10.16, 14.94)	12.58 (10.20, 14.34)	13.03 (9.80, 15.27)	12.76 (9.91, 15.81)
Mean solar zenith / degrees	45 (28, 59)	43 (25, 57)	40 (24, 56)	52 (30, 64)	51 (32, 64)	41 (24, 53)	41 (25, 56)
Mean hours since sunrise	6.27 (3.94, 8.62)	4.71 (2.76, 7.34)	3.13 (2.14, 4.79)	5.65 (3.94, 8.32)	8.58 (6.49, 10.19)	6.04 (4.26, 8.01)	5.23 (4.00, 6.97)
Rectal bleeding (baseline)							
Grade 0	832 (97%)	165 (97%)	48 (96%)	119 (98%)	255 (96%)	162 (98%)	83 (91%)
Grade 1	29 (3.4%)	5 (2.9%)	1 (2.0%)	2 (1.7%)	10 (3.8%)	3 (1.8%)	8 (8.8%)
Unknown	1 (0.1%)	0 (0%)	1 (2.0%)	0 (0%)	0 (0%)	0 (0%)	0 (0%)
Rectal bleeding (24m)							
Grade 0	770 (89%)	161 (95%)	42 (84%)	113 (93%)	245 (92%)	124 (75%)	85 (93%)
Grade 1	60 (7.0%)	4 (2.4%)	8 (16%)	5 (4.1%)	12 (4.5%)	27 (16%)	4 (4.4%)
Grade 2	28 (3.2%)	4 (2.4%)	0 (0%)	3 (2.5%)	8 (3.0%)	13 (7.9%)	0 (0%)
Grade 3	4 (0.5%)	1 (0.6%)	0 (0%)	0 (0%)	0 (0%)	1 (0.6%)	2 (2.2%)
Unknown	0 (0%)	0 (0%)	0 (0%)	0 (0%)	0 (0%)	0 (0%)	0 (0%)
Rectal bleeding (deterioration at 24m from baseline)	88 (10%)	9 (5.3%)	8 (16%)	8 (6.6%)	18 (6.8%)	40 (24%)	5 (5.5%)
Unknown	1	0	1	0	0	0	0
Diarrhoea (baseline)							
Grade 0	842 (98%)	164 (96%)	48 (96%)	120 (99%)	260 (98%)	163 (99%)	87 (96%)
Grade 1	17 (2.0%)	6 (3.5%)	1 (2.0%)	1 (0.8%)	3 (1.1%)	2 (1.2%)	4 (4.4%)
Grade 2	1 (0.1%)	0 (0%)	0 (0%)	0 (0%)	1 (0.4%)	0 (0%)	0 (0%)
Grade 3	0 (0%)	0 (0%)	0 (0%)	0 (0%)	0 (0%)	0 (0%)	0 (0%)
Unknown	2 (0.2%)	0 (0%)	1 (2.0%)	0 (0%)	1 (0.4%)	0 (0%)	0 (0%)
Diarrhoea (24m)							
Grade 0	796 (92%)	143 (84%)	42 (84%)	119 (98%)	257 (97%)	154 (93%)	81 (89%)
Grade 1	56 (6.5%)	23 (14%)	6 (12%)	2 (1.7%)	7 (2.6%)	9 (5.5%)	9 (9.9%)
Grade 2	8 (0.9%)	4 (2.4%)	2 (4.0%)	0 (0%)	1 (0.4%)	0 (0%)	1 (1.1%)
Unknown	2 (0.2%)	0 (0%)	0 (0%)	0 (0%)	0 (0%)	2 (1.2%)	0 (0%)
Diarrhoea (deterioration at 24m from baseline)	8 (0.9%)	4 (2.4%)	2 (4.0%)	0 (0%)	1 (0.4%)	0 (0%)	1 (1.1%)
Unknown	2	0	0	0	0	2	0
Urinary frequency (baseline)							
Grade 0	532 (62%)	129 (76%)	30 (60%)	106 (88%)	142 (54%)	79 (48%)	46 (51%)
Grade 1	311 (36%)	38 (22%)	17 (34%)	15 (12%)	116 (44%)	82 (50%)	43 (47%)
Grade 2	16 (1.9%)	3 (1.8%)	1 (2.0%)	0 (0%)	7 (2.6%)	3 (1.8%)	2 (2.2%)
Unknown	3 (0.3%)	0 (0%)	2 (4.0%)	0 (0%)	0 (0%)	1 (0.6%)	0 (0%)
Urinary frequency (24m)							
Grade 0	560 (65%)	121 (71%)	27 (54%)	85 (70%)	177 (67%)	90 (55%)	60 (66%)
Grade 1	274 (32%)	29 (17%)	22 (44%)	36 (30%)	87 (33%)	71 (43%)	29 (32%)
Grade 2	22 (2.6%)	20 (12%)	0 (0%)	0 (0%)	1 (0.4%)	0 (0%)	1 (1.1%)
Unknown	6 (0.7%)	0 (0%)	1 (2.0%)	0 (0%)	0 (0%)	4 (2.4%)	1 (1.1%)
Urinary frequency (deterioration at 24m from baseline)	159 (19%)	38 (22%)	11 (23%)	31 (26%)	36 (14%)	29 (18%)	14 (16%)
Unknown	9	0	3	0	0	5	1
Urinary incontinence (baseline)							
Grade 0	656 (76%)	112 (66%)	31 (62%)	97 (80%)	188 (71%)	149 (90%)	79 (87%)
Grade 1	144 (17%)	35 (21%)	10 (20%)	16 (13%)	62 (23%)	12 (7.3%)	9 (9.9%)
Grade 2	59 (6.8%)	23 (14%)	8 (16%)	8 (6.6%)	14 (5.3%)	3 (1.8%)	3 (3.3%)
Grade 3	2 (0.2%)	0 (0%)	0 (0%)	0 (0%)	1 (0.4%)	1 (0.6%)	0 (0%)
Unknown	1 (0.1%)	0 (0%)	1 (2.0%)	0 (0%)	0 (0%)	0 (0%)	0 (0%)
Urinary incontinence (24m)							
Grade 0	623 (72%)	84 (49%)	32 (64%)	103 (85%)	197 (74%)	127 (77%)	80 (88%)
Grade 1	157 (18%)	48 (28%)	11 (22%)	11 (9.1%)	51 (19%)	27 (16%)	9 (9.9%)
Grade 2	72 (8.4%)	33 (19%)	6 (12%)	7 (5.8%)	17 (6.4%)	7 (4.2%)	2 (2.2%)
Grade 3	6 (0.7%)	5 (2.9%)	1 (2.0%)	0 (0%)	0 (0%)	0 (0%)	0 (0%)
Unknown	4 (0.5%)	0 (0%)	0 (0%)	0 (0%)	0 (0%)	4 (2.4%)	0 (0%)
Urinary incontinence (deterioration at 24m from baseline)	131 (15%)	50 (29%)	8 (16%)	8 (6.6%)	29 (11%)	30 (19%)	6 (6.6%)
Unknown	5	0	1	0	0	4	0

[1]Median (Q1, Q3); n (%)
