# Supplementary material for: Circadian Genotypes in PER3 and Time of Prostate Cancer Radiotherapy Interact to Affect Risk of Late Side Effects
Source: Clin Cancer Res. 2026 Jul 13;32(19):4384–95. doi: 10.1158/1078-0432.CCR-26-0268 (PMC13628092; doi:10.1158/1078-0432.CCR-26-0268)
Supplement: Supplementary file 2 [file ccr-26-0268_supplementary_table_s2_suppts2.pdf]

Supplementary table 2a: Rectal bleeding base models

	Fixed effects			Mixed effects		
**Characteristic**	**OR**	**95% CI**	**p-value**	**OR**	**95% CI**	**p-value**
s.d treatment time	1.61	1.26, 2.05	**<0.001**	1.39	1.03, 1.87	**0.029**
BMI kg / m²	0.91	0.86, 0.97	**0.003**	0.92	0.87, 0.98	**0.008**
BED_{late}	1.00	0.99, 1.02	0.5	1.02	1.00, 1.03	0.066
Alpha blockers	2.40	1.28, 4.31	**0.004**	1.80	0.95, 3.43	0.073
Site.sd__(Intercept)				0.57		
AIC	545			533		
No. Obs.	860			860		
ROC-AUC	0.67			0.71		
ROC-AUC (CI 5%)	0.59			0.68		
ROC-AUC (CI 95%)	0.78			0.75		
ICC				0.09		

Abbreviations: CI = Confidence Interval, OR = Odds Ratio

Supplementary table 2b: Urinary incontinence base models

	Fixed effects			Mixed effects		
**Characteristic**	**OR**	**95% CI**	**p-value**	**OR**	**95% CI**	**p-value**
Radical prostatectomy	3.28	2.20, 4.90	**<0.001**	3.06	1.99, 4.72	**<0.001**
s.d. treatment time	1.61	1.25, 2.07	**<0.001**	1.38	1.02, 1.87	**0.037**
BMI kg / m²	1.02	0.98, 1.07	0.3	1.04	0.99, 1.09	0.13
BED_{late}	0.99	0.98, 1.00	0.2	0.99	0.98, 1.00	0.11
Site.sd__(Intercept)				0.47		
AIC	658			648		
No. Obs.	818			818		
ROC-AUC	0.68			0.72		
ROC-AUC (CI 5%)	0.58			0.67		
ROC-AUC (CI 95%)	0.73			0.75		
ICC				0.06		

Abbreviations: CI = Confidence Interval, OR = Odds Ratio
