# Supplementary material for: Circadian Genotypes in PER3 and Time of Prostate Cancer Radiotherapy Interact to Affect Risk of Late Side Effects
Source: Clin Cancer Res. 2026 Jul 13;32(19):4384–95. doi: 10.1158/1078-0432.CCR-26-0268 (PMC13628092; doi:10.1158/1078-0432.CCR-26-0268)
Supplement: Supplementary file 3 [file ccr-26-0268_supplementary_table_s3_suppts3.pdf]

Supplementary table 3a: Rectal bleeding (patients with deterioration from baseline at 12m but not 24m removed)

	rs11121022			rs172933			rs35128310			rs488728			rs696305			rs697689		
**Characteristic**	**OR**	**95% CI**	**p-value**	**OR**	**95% CI**	**p-value**	**OR**	**95% CI**	**p-value**	**OR**	**95% CI**	**p-value**	**OR**	**95% CI**	**p-value**	**OR**	**95% CI**	**p-value**
s.d. treatment time (hours)	1.78	1.30, 2.47	**<0.001**	1.74	1.27, 2.41	**<0.001**	1.71	1.25, 2.36	**<0.001**	1.73	1.26, 2.38	**<0.001**	1.74	1.27, 2.40	**<0.001**	1.76	1.28, 2.44	**<0.001**
BMI	0.87	0.81, 0.94	**<0.001**	0.88	0.82, 0.94	**<0.001**	0.88	0.82, 0.94	**<0.001**	0.88	0.82, 0.94	**<0.001**	0.87	0.81, 0.94	**<0.001**	0.87	0.81, 0.94	**<0.001**
BED_{late}	1.01	0.99, 1.03	0.4	1.01	0.99, 1.03	0.4	1.01	0.99, 1.03	0.4	1.01	0.99, 1.03	0.4	1.01	0.99, 1.03	0.4	1.01	0.99, 1.03	0.4
Alpha blocker	2.64	1.30, 5.12	**0.005**	2.85	1.40, 5.54	**0.003**	2.65	1.30, 5.16	**0.005**	2.65	1.31, 5.14	**0.005**	2.59	1.27, 5.06	**0.007**	2.91	1.42, 5.69	**0.002**
Treatment time (hours since sunrise)	1.18	1.03, 1.36	**0.017**	1.24	0.96, 1.61	0.10	1.20	0.97, 1.49	0.091	1.23	1.01, 1.50	**0.043**	0.90	0.79, 1.02	0.10	0.95	0.86, 1.06	0.4
SNP	2.68	1.20, 5.99	**0.016**	2.57	0.88, 8.16	0.10	2.35	0.90, 6.41	0.089	2.33	0.93, 6.07	0.076	0.36	0.14, 0.90	**0.033**	0.23	0.06, 0.77	**0.023**
Time * SNP	0.84	0.75, 0.95	**0.006**	0.88	0.76, 1.03	0.11	0.89	0.77, 1.02	0.093	0.87	0.76, 0.99	**0.039**	1.21	1.06, 1.39	**0.006**	1.22	1.04, 1.45	**0.018**
AIC	451			455			455			454			450			452		
No. Obs.	713			713			713			713			713			713		

Abbreviations: CI = Confidence Interval, OR = Odds Ratio. Event n = 74

Supplementary table 3b: Rectal bleeding (deterioration from baseline) at either 12m or 24m post-radiotherapy

	rs11121022			rs172933			rs35128310			rs488728			rs696305			rs697689		
**Characteristic**	**OR**	**95% CI**	**p-value**	**OR**	**95% CI**	**p-value**	**OR**	**95% CI**	**p-value**	**OR**	**95% CI**	**p-value**	**OR**	**95% CI**	**p-value**	**OR**	**95% CI**	**p-value**
s.d. treatment time (hours)	1.79	1.38, 2.32	**<0.001**	1.75	1.36, 2.28	**<0.001**	1.75	1.35, 2.27	**<0.001**	1.75	1.35, 2.27	**<0.001**	1.74	1.35, 2.27	**<0.001**	1.76	1.36, 2.28	**<0.001**
BMI	0.91	0.86, 0.96	**0.001**	0.92	0.87, 0.97	**0.002**	0.92	0.87, 0.97	**0.002**	0.92	0.87, 0.97	**0.001**	0.91	0.86, 0.96	**0.001**	0.92	0.87, 0.96	**0.001**
BED_{late}	1.00	0.99, 1.01	0.9	1.00	0.99, 1.01	>0.9	1.00	0.99, 1.01	0.9	1.00	0.99, 1.01	>0.9	1.00	0.99, 1.01	0.9	1.00	0.99, 1.01	>0.9
Alpha blocker	2.60	1.46, 4.49	**<0.001**	2.61	1.47, 4.53	**<0.001**	2.59	1.46, 4.48	**<0.001**	2.58	1.45, 4.47	**<0.001**	2.52	1.42, 4.37	**0.001**	2.65	1.49, 4.62	**<0.001**
Treatment time (hours since sunrise)	1.09	0.98, 1.22	0.12	1.05	0.87, 1.28	0.6	1.08	0.91, 1.27	0.4	1.09	0.93, 1.27	0.3	0.92	0.83, 1.02	0.13	0.96	0.88, 1.05	0.4
SNP	1.77	0.91, 3.44	0.092	1.22	0.55, 2.81	0.6	1.44	0.68, 3.11	0.3	1.46	0.70, 3.06	0.3	0.57	0.27, 1.17	0.13	0.53	0.20, 1.31	0.2
Time * SNP	0.90	0.81, 0.99	**0.036**	0.96	0.86, 1.08	0.5	0.94	0.84, 1.06	0.3	0.93	0.84, 1.04	0.2	1.12	1.00, 1.25	**0.047**	1.10	0.97, 1.25	0.2
AIC	639			643			643			642			639			642		
No. Obs.	821			821			821			821			821			821		

Abbreviations: CI = Confidence Interval, OR = Odds Ratio. Event n = 116
