# Supplementary material for: Circadian Genotypes in PER3 and Time of Prostate Cancer Radiotherapy Interact to Affect Risk of Late Side Effects
Source: Clin Cancer Res. 2026 Jul 13;32(19):4384–95. doi: 10.1158/1078-0432.CCR-26-0268 (PMC13628092; doi:10.1158/1078-0432.CCR-26-0268)
Supplement: Supplementary file 4 [file ccr-26-0268_supplementary_table_s4_suppts4.pdf]

## **Supplementary table 4a:** Urinary incontinence (patients with toxicity at 12m but not 24m removed)

| | rs172933 | | | rs696305 | | | rs488728 | | | rs228640 | | | rs697689 | | |
|---|---|---|---|---|---|---|---|---|---|---|---|---|---|---|---|
| **Characteristic** | **OR** | **95% CI** | **p-value** | **OR** | **95% CI** | **p-value** | **OR** | **95% CI** | **p-value** | **OR** | **95% CI** | **p-value** | **OR** | **95% CI** | **p-value** |
| Radical prostatectomy | 3.36 | 2.22, 5.12 | **<0.001** | 3.48 | 2.30, 5.29 | **<0.001** | 3.43 | 2.27, 5.22 | **<0.001** | 3.45 | 2.28, 5.25 | **<0.001** | 3.44 | 2.27, 5.24 | **<0.001** |
| s.d. treatment time | 1.67 | 1.29, 2.17 | **<0.001** | 1.60 | 1.24, 2.07 | **<0.001** | 1.61 | 1.24, 2.08 | **<0.001** | 1.62 | 1.25, 2.10 | **<0.001** | 1.65 | 1.27, 2.14 | **<0.001** |
| BMI | 1.03 | 0.98, 1.07 | 0.3 | 1.03 | 0.98, 1.07 | 0.3 | 1.03 | 0.98, 1.07 | 0.3 | 1.02 | 0.98, 1.07 | 0.3 | 1.03 | 0.98, 1.07 | 0.3 |
| $BED_{late}$ | 0.99 | 0.98, 1.00 | 0.10 | 0.99 | 0.98, 1.00 | 0.15 | 0.99 | 0.98, 1.00 | 0.11 | 0.99 | 0.98, 1.00 | 0.12 | 0.99 | 0.98, 1.00 | 0.11 |
| Time (hours from solar 12:00) | 0.49 | 0.32, 0.73 | **<0.001** | 1.13 | 0.94, 1.35 | 0.2 | 0.62 | 0.45, 0.84 | **0.002** | 0.76 | 0.63, 0.93 | **0.008** | 1.09 | 0.93, 1.28 | 0.3 |
| SNP | 0.44 | 0.24, 0.82 | **0.009** | 1.85 | 1.07, 3.18 | **0.026** | 0.50 | 0.29, 0.87 | **0.013** | 0.47 | 0.24, 0.87 | **0.019** | 2.39 | 1.23, 4.60 | **0.009** |
| Time * SNP | 1.53 | 1.20, 1.96 | **<0.001** | 0.75 | 0.61, 0.92 | **0.006** | 1.38 | 1.13, 1.71 | **0.002** | 1.40 | 1.13, 1.75 | **0.003** | 0.67 | 0.51, 0.86 | **0.002** |
| AIC | 623 | | | 629 | | | 627 | | | 627 | | | 626 | | |
| BIC | 660 | | | 666 | | | 663 | | | 664 | | | 663 | | |
| No. Obs. | 748 | | | 748 | | | 748 | | | 748 | | | 748 | | |

Abbreviations: CI = Confidence Interval, OR = Odds Ratio. Event n = 123

## **Supplementary table 4b:** Urinary incontinence, deterioration from baseline at either 12m or 24m

| | rs172933 | | | rs696305 | | | rs488728 | | | rs228640 | | | rs697689 | | |
|---|---|---|---|---|---|---|---|---|---|---|---|---|---|---|---|
| **Characteristic** | **OR** | **95% CI** | **p-value** | **OR** | **95% CI** | **p-value** | **OR** | **95% CI** | **p-value** | **OR** | **95% CI** | **p-value** | **OR** | **95% CI** | **p-value** |
| Radical prostatectomy | 2.68 | 1.87, 3.84 | **<0.001** | 2.79 | 1.95, 4.01 | **<0.001** | 2.74 | 1.91, 3.93 | **<0.001** | 2.74 | 1.92, 3.93 | **<0.001** | 2.74 | 1.91, 3.93 | **<0.001** |
| s.d. treatment time | 1.52 | 1.22, 1.91 | **<0.001** | 1.49 | 1.19, 1.86 | **<0.001** | 1.50 | 1.20, 1.87 | **<0.001** | 1.50 | 1.20, 1.88 | **<0.001** | 1.51 | 1.21, 1.90 | **<0.001** |
| BMI | 1.01 | 0.97, 1.05 | 0.7 | 1.01 | 0.97, 1.05 | 0.7 | 1.01 | 0.97, 1.05 | 0.7 | 1.01 | 0.97, 1.05 | 0.7 | 1.01 | 0.97, 1.05 | 0.7 |
| $BED_{late}$ | 0.99 | 0.98, 1.01 | 0.2 | 0.99 | 0.98, 1.01 | 0.3 | 0.99 | 0.98, 1.01 | 0.2 | 0.99 | 0.98, 1.01 | 0.3 | 0.99 | 0.98, 1.01 | 0.2 |
| Time (hours from solar 12:00) | 0.66 | 0.47, 0.92 | **0.016** | 1.23 | 1.05, 1.44 | **0.010** | 0.73 | 0.56, 0.95 | **0.020** | 0.88 | 0.74, 1.03 | 0.12 | 1.17 | 1.01, 1.34 | **0.031** |
| SNP | 0.57 | 0.33, 0.99 | **0.043** | 1.90 | 1.16, 3.10 | **0.011** | 0.56 | 0.34, 0.91 | **0.019** | 0.49 | 0.27, 0.86 | **0.014** | 2.11 | 1.15, 3.82 | **0.015** |
| Time * SNP | 1.33 | 1.09, 1.63 | **0.005** | 0.74 | 0.62, 0.89 | **0.001** | 1.30 | 1.09, 1.55 | **0.004** | 1.30 | 1.08, 1.58 | **0.006** | 0.70 | 0.56, 0.87 | **0.002** |
| AIC | 803 | | | 800 | | | 803 | | | 804 | | | 801 | | |
| BIC | 840 | | | 838 | | | 840 | | | 841 | | | 838 | | |
| No. Obs. | 818 | | | 818 | | | 818 | | | 818 | | | 818 | | |

Abbreviations: CI = Confidence Interval, OR = Odds Ratio. Event n = 171
