# Supplementary material for: Circadian Genotypes in PER3 and Time of Prostate Cancer Radiotherapy Interact to Affect Risk of Late Side Effects
Source: Clin Cancer Res. 2026 Jul 13;32(19):4384–95. doi: 10.1158/1078-0432.CCR-26-0268 (PMC13628092; doi:10.1158/1078-0432.CCR-26-0268)
Supplement: Supplementary file 5 [file ccr-26-0268_supplementary_appendix_s1_suppas1.docx]

# Additional REQUITE Consortium Collaborators

REQUITE Consortium collaborators in addition to those listed as authors on the main manuscript.

## Department of Radiation Oncology, Ghent University Hospital, Belgium

Piet Ost, Yolande Lievens, Pieter Deseyne, Fréderic Duprez, Marc van Eijkeren, Elhaseen Elhamin, Valérie Fonteyne, Annick Van Greveling, Marcus Mareel, Christel Monten, Wilfried De Neve, Leen Paelinck, Giselle Post, Martijn Swimberghe, Katrien Vandecasteele, Tom Vercauteren.

## Department of Radiation Oncology, University Hospitals Leuven, Belgium

Rita Aerts, Soumia Arredouani, Gilles Defraene, Erik van Limberghen, Stephanie Peeters, Caroline Weltens, Karin Haustermans, Maarten Lambrecht.

## Patient Advocates

Erik Briers (Patient Advocate, Hasselt, Belgium); Hilary Stobart (Independent Cancer Patients’ Voice, UK).

## Maastricht University Medical Center, Department of Radiation Oncology (Maastro Clinic), Maastricht, The Netherlands

Ben Vanneste.

## Department of Radiation Oncology, University Federation of Radiation Oncology, Montpellier Cancer Institute, Univ Montpellier MUSE, Inserm U1194, Montpellier, France

Celine Bourgier, Muriel Brengues, Roxana Draghici, David Azria, Marie-Pierre Farcy-Jacquet.

## Division of Cancer Epidemiology, German Cancer Research Center (DKFZ), Heidelberg, Germany

Irmgard Helmbold, Anusha Müller, Jenny Chang-Claude, Philipp Heumann

## Department of Radiation Oncology, Universitätsmedizin Mannheim, Medical Faculty Mannheim, Heidelberg University, Mannheim, Germany

Michael Ehmann, Benjamin Gauter-Fleckenstein, Frank Giordano, Carsten Herskind, Marlon Veldwijk.

## Fondazione IRCCS Istituto Nazionale dei Tumori, Radiotherapy Department, Via G Venezian 1, Milan, Italy

Marzia Franceschini

## Vall d’Hebron Institute of Oncology (VHIO), Vall d’Hebron, Barcelona Hospital Campus, Cellex Center, Lab 2.02A. c/ Natzaret, 115-117 08035 Barcelona, Spain

Alexandra Giraldo, Mónica Ramos, Meritxell Mollà

## Fundación Pública Galega de Medicina Xenómica / Instituto de Investigación Sanitaria de Santiago de Compostela, Santiago de Compostela, Spain

Ana Crujeiras González, Belinda Rodríguez-Lage, Juan Fernández-Tajes, Antonio Gómez-Caamaño

## Department of Radiation Oncology, Hospital Clínico Universitario de Santiago de Compostela, SERGAS, Santiago de Compostela, Spain

Patricia Calvo-Crespo, Angel Carracedo, Monserrat Miranda Currás, Begoña Taboada-Valladares, Irene Fajardo-Paneque, Paloma Sosa-Fajardo, Laura Torrado-Moya, Isabel Dominguez-Rios, Ana Carballo, Paula Peleteiro.

## Translational Radiobiology Group, University of Manchester / Christie Hospital, Manchester, United Kingdom

Rebecca M. Elliott, Corinne Faivre-Finn

## Department of Genetics and Genome Biology / Leicester Cancer Research Centre, University of Leicester, Leicester, United Kingdom

Hannah Dobbelaere, Kerstie Johnson, Samuel Lavers, Vicki Emms

## Centre for Cancer Genetic Epidemiology, University of Cambridge, United Kingdom

Alison Dunning, Laura Fachal.
